# Modeling human cancer-related regulatory modules by GA-RNN hybrid algorithms

**DOI:** 10.1186/1471-2105-8-91

**Published:** 2007-03-14

**Authors:** Jung-Hsien Chiang, Shih-Yi Chao

**Affiliations:** 1 Department of Computer Science and Information Engineering, National Cheng Kung University, Tanian, Taiwan

## Abstract

**Background:**

Modeling cancer-related regulatory modules from gene expression profiling of cancer tissues is expected to contribute to our understanding of cancer biology as well as developments of new diagnose and therapies. Several mathematical models have been used to explore the phenomena of transcriptional regulatory mechanisms in *Saccharomyces cerevisiae*. However, the contemplating on controlling of feed-forward and feedback loops in transcriptional regulatory mechanisms is not resolved adequately in *Saccharomyces cerevisiae*, nor is in human cancer cells.

**Results:**

In this study, we introduce a Genetic Algorithm-Recurrent Neural Network (GA-RNN) hybrid method for finding feed-forward regulated genes when given some transcription factors to construct cancer-related regulatory modules in human cancer microarray data. This hybrid approach focuses on the construction of various kinds of regulatory modules, that is, Recurrent Neural Network has the capability of controlling feed-forward and feedback loops in regulatory modules and Genetic Algorithms provide the ability of global searching of common regulated genes. This approach unravels new feed-forward connections in regulatory models by modified multi-layer RNN architectures. We also validate our approach by demonstrating that the connections in our cancer-related regulatory modules have been most identified and verified by previously-published biological documents.

**Conclusion:**

The major contribution provided by this approach is regarding the chain influences upon a set of genes sequentially. In addition, this inverse modeling correctly identifies known oncogenes and their interaction genes in a purely data-driven way.

## Background

A regulatory module is a set of genes that is regulated or co-regulated by one or more common transcription factors (TFs). A TF is a protein that binds to a cis-regulatory element (e.g. an enhancer, a TATA box) and thereby, directly or indirectly, positively or negatively affects the initiation of transcription of regulated genes. A cancer-related regulatory module is a set of genes (oncogenes or tumor suppressor genes) that is regulated by one ore more common TFs. Modeling the cancer-related regulatory modules of the cell division cycle in human cells is a critical and fundamental step toward understanding cancers. The aim of this paper is not only to drive cancer-related regulatory modules, but also to identify the relationships of regulations between genes that fit the feed-forward or feedback influences. A feed-forward regulatory module, contains a TF that controls a second TF at later time points and has the additional feature that both TFs bind to common target genes. Therefore, the major contribution of this study is regarding the chain influences upon a set of genes sequentially. That is, to construct a simple cancer-related regulatory pathway with feedback loop and feed-forward controlled relationships achieved by modified Recurrent Neural Network (RNN) architecture [1]. Combining modified multi-layer RNN with the global searching ability of Genetic Algorithms (GA) [2], this approach can efficiently select regulated target genes as well. We also provide the solution of analysis time-course gene expression data. For example, one particular TF expressed highly in S/G1 phase may regulate its target genes expressed highly in the M (mitotic) phase. That is, our modified GA-RNN hybrid algorithm has the capability of finding target regulated genes at a later time point (e.g. *t *+ 2) when given a TF at an earlier time point (e.g. *t*).

### Machine learning approaches to microarray analysis

There are many types of gene transcriptional regulatory related approaches which have been proposed in the past. Their nature and composition are categorized by several factors: considering gene expression values [3,4], the causal relationship between genes, e.g. with Bayesian analysis or Dynamic Bayesian Networks[5,6], and the time domain e.g. discrete or continuous time [7-10]. The genome-wide transcriptional program during the cell cycle has been investigated in a wide range of organisms, including yeast [11], bacteria [12], primary human fibroblasts [13,14], and human HeLa cells [15]. However, consideration of feedback and feed-forward control in regulatory modules is also important. That is, some genes have unique characteristics, for instance, they regulate themselves or they regulate genes in the following further time points. Unfortunately, constructing regulatory modules with feedback and feed-forward controls is not mentioned by [3-6]. Moreover, genes may have one or more activators or inhibitors which co-regulate the transcription levels of genes in regulatory modules. Lots of the cell's activities are organized as sets of genes co-regulated by some particular TFs to respond to different conditions. Therefore, the present challenge is to understand how transcription factors control global gene expression programs, i.e., specific gene expression programs involve regulated transcription of many other genes in different time points or involve regulated transcription of themselves. Our approach aims to provide a system to construct regulatory modules with feedback and feed-forward control mechanisms that illustrate cancer-related genes and their cause-effect relations to other genes.

### Recent approaches to gene expression of human cancers

Gene expression profiling has been widely used for cancer research. The results provided by [16] and [17] have shown that co-expression of gene pairs in multiple data sets are correlated with functional relatedness. According to [16], they seek pairs of genes based on the correlation of their expression profiles in multiple data sets, and define these pairs of genes reliably co-expressed to establish a high-confidence network of more than 8,000 genes connected by co-expression links that are observed in the data sets. However, global co-expression patterns have not been determined for cancer, and it is still unknown what are the key genes or gene groups that have been causing or stabilizing the observed global cancer-related patterns. Likewise, it is of interest to know which genes are the factors that initiate the regulation to another possibly pathological state. Hence, we try to deal with this problem by identifying genes whose regulatory functions have interventions in the global cancer-related gene expression profiles.

## Results

### Results of human cancer data

The human cell cycle data set consists of almost 30,000 genes and over 44 time points for each of the experimental data. As a result, the number of gene combinations is more significant than the yeast data set. We list some experimental results in Table 4. In these experiments, the number of epochs of RNN is 200, and the number of generations of GA is varied while recording the error rates for the training data. The minimum value of RMSE decreases as the number of GA generation increases.

**Table 4:** The experimental results of GA with RNN for human cell cycle data

**GA generations**	**Average RMSE**	**The minimum RMSE**
100	5.34	2.78
500	3.47	1.86
1000	1.96	0.55
1500	1.31	0.38
2000	0.84	0.17
2500	0.81	0.16

Notice that the minimum RMSE for GA generations 2000 and 2500 are 0.17 and 0.16, respectively. Comparing these two experimental results, although the number of GA generations is supplementary, there is no conspicuous diminishing for the value of RMSE. Moreover, the maximum number of GA generations for the yeast data set is less than half of the human data set. Searching for "good" combinations of regulated target genes from the human data set yields much more permutations than yeast.

**Figure 4:**
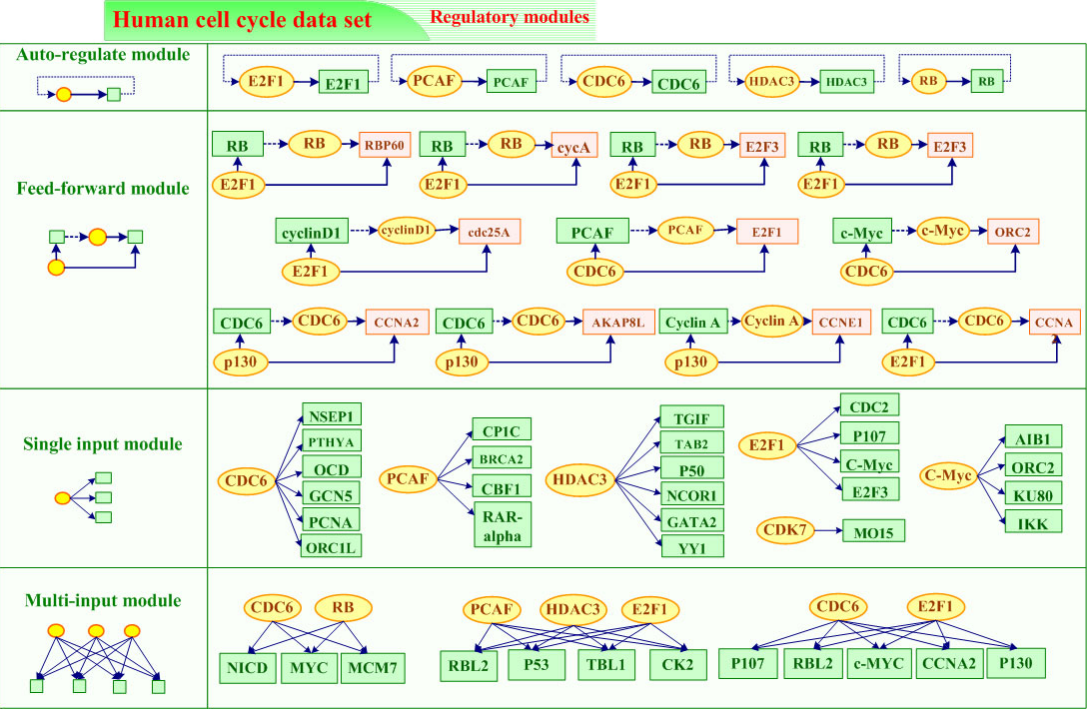
**Some experimental results of regulatory modules for human cell cycle data set**. Circles indicate TFs and squares represent genes. Solid arrows indicate regulation relationships between TFs and their target genes.

We also demonstrate some relationships of cancer-related regulation in Figure 4. The E2F1 gene has been biologically proven a key regulator of the cell cycle. As noted by Stanelle *et al*. [18], the p16/RB/E2F regulatory pathway, which controls transit through the G1 restriction point of the cell cycle, is one of the most frequent targets of genetic alterations in human cancer. Likewise, Figure 4 shows the same idea of the E2F1 and RB regulatory relationship, and also proves the ability of our system. It is also known that, E2F1 participates in the progress of apoptosis by regulating p53, and this is indicated in Figure 4 as well. P53 is a tumor suppressor whose inactivation is observed in most human cancers [19]. P53 also plays a central role in regulating cell growth, particularly in response to various forms of stress, including DNA damage and viral infection, because p53 sits on a critical node of signal transduction networks that control cell growth and death. Figure 4 illustrates that PCAF is a co-activator of p53 transcription identified by our system, which is biologically supported by Zhao *et al*. [20]. The same as CDC6, PCAF is identified as an auto-regulated TF, moreover, it up-regulates the NICD, CBF1, and BRCA2 target genes. More results and biological evidences are provided in additional file 1.

### Compare human cancer data results to yeast cell cycle

An example of periodically expressed gene in human cells but not in yeast is the human CDK7 (homologous of *S. cerevisiae *KIN28). Yeast KIN28 is not found as a TF at the transcriptional level, but the CDK7 is a TF that regulates MO15. Human gene CDC6 is homologous of *S. cerevisiae *CDC6, which is identified by our system, and is an auto-regulated and crucial TF to GCN5, pRB, and NICD genes while yeast CDC6 is not. The most interesting and complicated gene of the human data set that system came out is E2F1. It is likely that some of the periodically expressed genes in human cell that do not have periodically expressed correspondents in yeast are subject to multiple layers of regulation. It is also reasonable that, multi-layer regulation in human cell, such as the phosphorylation and proteolysis, are known for some well-studied cell cycle genes. Therefore, the regulatory module starting on E2F1 TF shows the chain processes of the regulatory mechanism, which implies the complication of human cancer-related regulatory manipulations. One known gene, p53, also represents an intriguing consequence. After inputting HDAC3 TF to the GA-RNN algorithm, some negative values are reported by the neural network weight matrix, which indicates inhibitor targets. It appears that there is a similar case, the GATA2, down-regulated by HDAC3 as well, and both are confirmed by Juan *et al*. [21] and Ozawa *et al*. [22]. These listed genes are strongly expressed in proliferative tumors, and have regulatory relationships with other genes. This may further prove to be a useful source of additional drug targets of this kind. On the other hand, most of the genes identified by our system periodically expressed in both species are involved in DNA replication, DNA repair, DNA metabolism and mitosis. In the viewpoint of biology, these genes involved in mitosis or DNA replication, in all probability, have connections with cancer diseases.

**Figure 5:**
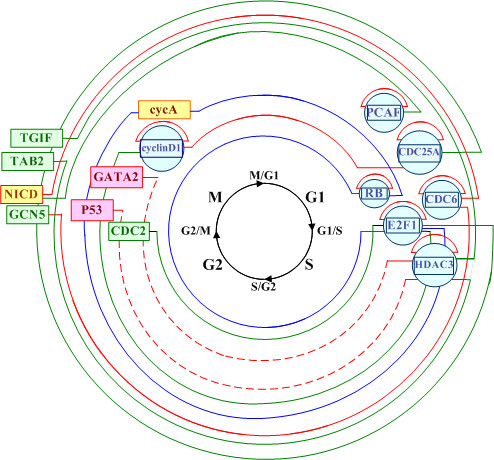
**Some experimental results of regulatory modules for human cell cycle data set**. The green lines indicate regulatory connections predicted by this approach. The red lines indicate regulatory connections that predicted by this approach and also are confirmed by biological experiments. The red dotted lines represent the negative regulatory controlling. The blue lines shown in this graph demonstrate the feed-forward controlling between E2F1, RB and the target gene cycA. All TFs are represented by blue circles and the squares indicate the target genes.

We also show some experimental results with G1/S/G2/M phases in figure 5. The green lines shown in figure 5 indicate regulatory connections predicted by this approach. The red lines indicate regulatory connections that predicted by this approach and also are confirmed by biological experiments. The red dotted lines represent the negative regulatory controlling, which are also confirmed by biological experiments. The blue lines shown in figure 5 demonstrate the feed-forward controlling between E2F1, RB and the target gene cycA. It is clear that the E2F1 is a start TF and controls the second TF, RB. Both E2F1 and RB regulate the target gene, cycA, which form a feed-forward regulatory motif.

## Discussion

The construction of cancer-related regulatory modules from temporal expression data is one of the most important problems in computational biology. It is acknowledged that the causes of heterogeneity genetic-related circumstances, such as the cell cycle, or cancer diseases, are products of complex interactions between genes over time. The analysis of cancer-related gene expression data will thus become increasingly widespread. When appraising approaches for discovery of cancer-related regulatory modules, the amount and type of sources of data must be taken into account. Besides, the approach must be capable of handling noisy and high dimensional gene expression data. The approach described here has been shown to be effective with real-world expression data. The stochastic nature of GA means that the same results can not be expected from each run of the algorithm, and the GA is run for a fixed number of generations for each output of regulatory modules. However, to increase the number of genes that the GA can select from, it could require more GA generations. As a result, increasing the GA generations also increases the computational time, although it does show that results on microarray data can be discovered correctly by the GA used in our approach. In addition, this approach builds modules "piece by piece", that is, regulatory module by regulatory module. Imagine that the network motif described in section 2 is one of the transcriptional regulatory mechanism units with a specific set of genes, including the influences and the targets. We discover all the formed units one by one and eventually join these units by their simultaneously existing TFs. The above-mentioned contents are the advantages of generating smaller but more precise regulatory modules, in that each of the paths or the units (or genes) in the modules can be seen without being masked by other connections. It is not the same as traditional complicated regulatory relationships, which are too many to visualize as a network to yield useful information in a digestible format for biologists.

### Compare to related researches

The ordering of regulatory processing can be displayed faithfully by this approach, especially the feed-forward motif, which represents a very simple regulator pathway. The phenomenon of this biology mechanism is often seen but ignored when constructing regulatory modules. Bayesian Networks have been proven to be an efficient methodology to reveal the cause-effect relationships from microarray experimental data, but may be deficient in dealing with the control of feedback or feed-forward issues. A major contribution provided by this approach is the discovery of not only the cause-effect relationships between genes but also the chain influences upon a set of genes sequentially. The mechanism of chain influences is achieved by modified RNN architecture, which includes the nonlinear mapping capability and the ability of time delay in constructing regulatory modules. The other advantage of this study is the power of global searching for regulated target genes provided by GA. Those selected target genes are judged whether they are "true target genes" or not according to the RMSE provided by RNN, and the results substantiate the capability of our hybrid GA-RNN method. Compared to [5] (see additional file 2), this paper limits potential regulators to those genes with either earlier or simultaneous expression changes (up- or down-regulation) in relation to their target genes, which we also achieve the results. Moreover, we provide feedback and feed-forward regulation control relations between transcription factors and their targets, that is, we contribute more complicated regulatory relationships of regulatory modules.

Compared to Keedwell *et al*. [3], which uses a supervised single-layer artificial neural network (ANN) to construct the regulatory connections between genes, the first improvement of our approach is that we use modified multi-layer RNN to complete the feed-forward, feedback, auto-regulate, and multi-input regulatory modules, which means we show clearly the transcriptional regulatory mechanisms. Secondly, Keedwell *et al*. [3] provided most of the significant connections in the network by repeated GA. In our approach, we intersect the regulatory modules that output from different GA generations. Take Table 4 as an example, the GA generations for human cell cycle data are 100, 500, 1000, 1500, 2000, and 2500, respectively. We collect the repeated regulatory modules that are appeared in 1000, 1500, 2000, and 2500 generations. It means that the significant regulatory modules are aggregated. Finally, Keedwell *et al*. construct their regulatory connections by microarray expression values; nevertheless, we also consider the transcription factor binding sites sequences, which consider more biological factors while constructing regulatory modules. We also provide more biological evidences for human cell cycle data, and the results compared with [7] are listed in additional file 1.

## Conclusion

We combine the GA and RNN computing approaches to construct the cancer-related regulatory modules in *silico*. Upon the microarray data and the sequences of transcription factor binding sites, the approach has been shown to be able to accurately fit the data on which it is trained. We also observe that some TFs play critical roles in various motifs. In other words, some functions of TFs are fit for several kinds of regulatory modules. We then adopt these characteristics by training the RBF classifier [1] for categorizing TFs. Additionally, the experimental results have proven that the GA-RNN hybrid algorithm has the capability of constructing the feedback and feed-forward regulatory modules. RNNs with diversified architectures indicate varied regulatory mechanisms to construct complete regulatory modules with feedback and feed-forward controls. Combining modified RNN with GA, it provides the global searching capacities to find proper target regulated genes for some TFs. The chromosomes that the GA used are combinations of target genes and the crossover and mutation operators used by GA on all chromosomes alter the choice of output gene combinations. This approach is on the basis of both gene expression data and sequences data, so it is time significant and binding region significant data analysis. Summing up, since this method has been previously shown to also classify TFs as well and then construct regulatory modules, it can be considered a candidate multipurpose tool for microarray expression data analysis.

## Methods

### Human cell cycle microarray data

Microarray time course measurement of genome-wide mRNA expression levels allows genome-wide prediction of cell division cycle regulated genes. In each cell division cycle, cells pass through four phases, namely, M-G2-S-G1, in a fixed order. Each cell division cycle regulates gene expressions in one of these phases, and results in a rise in the possibility that the cell division cycle regulated genes would reveal periodic expressions if they are studied for more than one cycle. This phenomenon is the basis for detecting genes with oscillated expressions in synchronized cell culture to discover the cell division cycle regulated genes. Data from Whitfield *et al*. [23] is downloaded from the reference web site. The genome-wide program of gene expression during the cell division cycle in a human cancer cell line (HeLa) is characterized using cDNA microarrays. The goal of human cell cycle analysis from Whitfield *et al*. is to identify >850 genes periodically expressed during the cell cycle, and to show that most of these genes have been previously associated with the proliferation of tumors during the human cell division cycle as well. The data in this report provide a comprehensive catalog of cell cycle regulated genes that can serve as a starting point for functional discovery. We adopt this data set to construct cancer-related regulatory modules with feedback or feed-forward controlled target genes which are regulated by some specific TFs.

### Yeast cell cycle data

The data of Spellman *et al*. [24] is downloaded from the reference web site. We use this data set to construct regulatory modules and target genes which are also regulated by some specific TFs. Since this data set has been used to construct regulatory modules by various approaches in the past, we regard it as the testing data set to prove the efficiency of our approach. The experimental results and biological supports are provided in additional file 2 and additional file 3.

### Transcription factor binding sites

Our computational method attempts to integrate gene expression data and sequence data. Transcription factors bind short DNA motifs, namely, the transcription factor binding site, which plays a central role in recruiting the transcriptional mechanism at the promoters of genes and leading to the initiation of their transcription. As a result, we collect known transcription factor binding sites sequences from ENSEMBL [25], TRANSFAC [26], SGD [27] and YPD [28] for human and yeast, which are listed in additional file 4.

### The network motifs

According to Lee *et al*. [29], there are five network motifs for transcriptional regulatory modules. We provide the detail descriptions in additional file 5.

### Pre-processing

With all the materials that described above, we next perform the data pre-processing procedure. For instance, microarray data with missing values that occurred at certain time points for some genes have been deleted from the data set, since we can not appraise what the real values are. Besides, all microarray numeric data are re-scaled between 0 and 1 by divided the maximum value for each gene. All the transcription factor binding sites queried from databases are originally represented by sequences of amino acids and encoded into numeric codes which are listed in Table 1.

**Table 1:** The encoded numbers of amino acids

Amino acids	A	G	C	T	P	R	S	W	D	E	F	I	K	L	N	Q	V	H	Y	M
Encoded number	0.05	0.1	0.15	0.2	0.25	0.3	0.35	0.4	0.45	0.5	0.55	0.6	0.65	0.7	0.75	0.8	0.85	0.9	0.95	1

### Categorize human cell cycle-related transcription factors by RBF

Whitfield *et al*. [23] have identified 874 genes that show periodic expression across the human cell cycle in a well-studied cancer cell line (HeLa). However, not all the 874 genes are TFs; our approach is intended to reveal the target genes that are regulated by one particular TF. To achieve this, we search GO (Gene Ontology) terms for 874 genes from the GO web site [30]. Only genes with GO annotation terms, such as "transcription factor activity", "transcription factor complex", "regulation of cell cycle" and so on, are kept. Genes without related transcriptional function GO terms are left behind. In an analogous manner, we regroup TFs for Homo sapiens. Take transcription factor E2F1 as an example, a list of reactions which go out from E2F1 that representing E2F1 is served as a signal donor. These downstream reactions contain E2F1 itself, which cause E2F1 to fit the definition of an auto-regulating TF. What is more, the expression of most E2F1-dependent genes, such as P107 and RB1, peaks at the G1/S boundary. Additionally, E2F1 is also involved in regulating genes that control other phases of the cell cycle. Such as cycA and cdc2, whose expression remain high throughout the S-phase and into the G2 phase, which characterizes E2F1 as a single-input, multi-input and feed-forward motif factor [31]. As a result, we regroup human sapiens TFs into several catalogs according to biological documents and list the classified samples in Table 2.

**Table 2:** Some examples of regrouped TFs for Homo sapiens

**Gene**	**Single Input**	**Multi Input**	**Feed-forward**	**Auto-regulate**	**Encoded catalog number of RBF classifier**
E2F1	√	√	√	√	00
CDC6	√	√	√	√	00
HDAC3	√	√		√	10
CDK7	√	√			11

Under this approach, the RBF network architecture is one input layer with two kinds of input data, one hidden layer, and one output layer, which is illustrated in additional file 6.

### Construction of regulatory modules

RNNs are neural networks with one or more feedback loops. Given a multilayer perceptron as the basic building block, we may have feedback from the output neurons of the multilayer perceptron to the input layer. Similarly, the architectures of gene regulatory modules also have feedback from the target genes to TFs, which represent positive or negative effects on those genes that influence themselves. When the multilayer perceptron has two or more hidden layers, the possible forms of global feedback expand even further, such as the feed-forward motif. We train different RNN architectural layouts for various network modules, as described in the material section.

**Figure 1:**
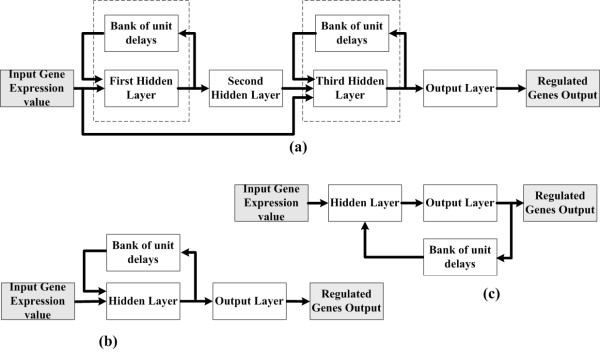
**RNN architectures for each different kind of regulatory module module used by this approach**. (a) is represented for gene feed-forward regulatory modules, (b) is represented for auto-regulate modules, and (c) is represented for single-input and multi-input regulatory modules.

**Figure 2:**
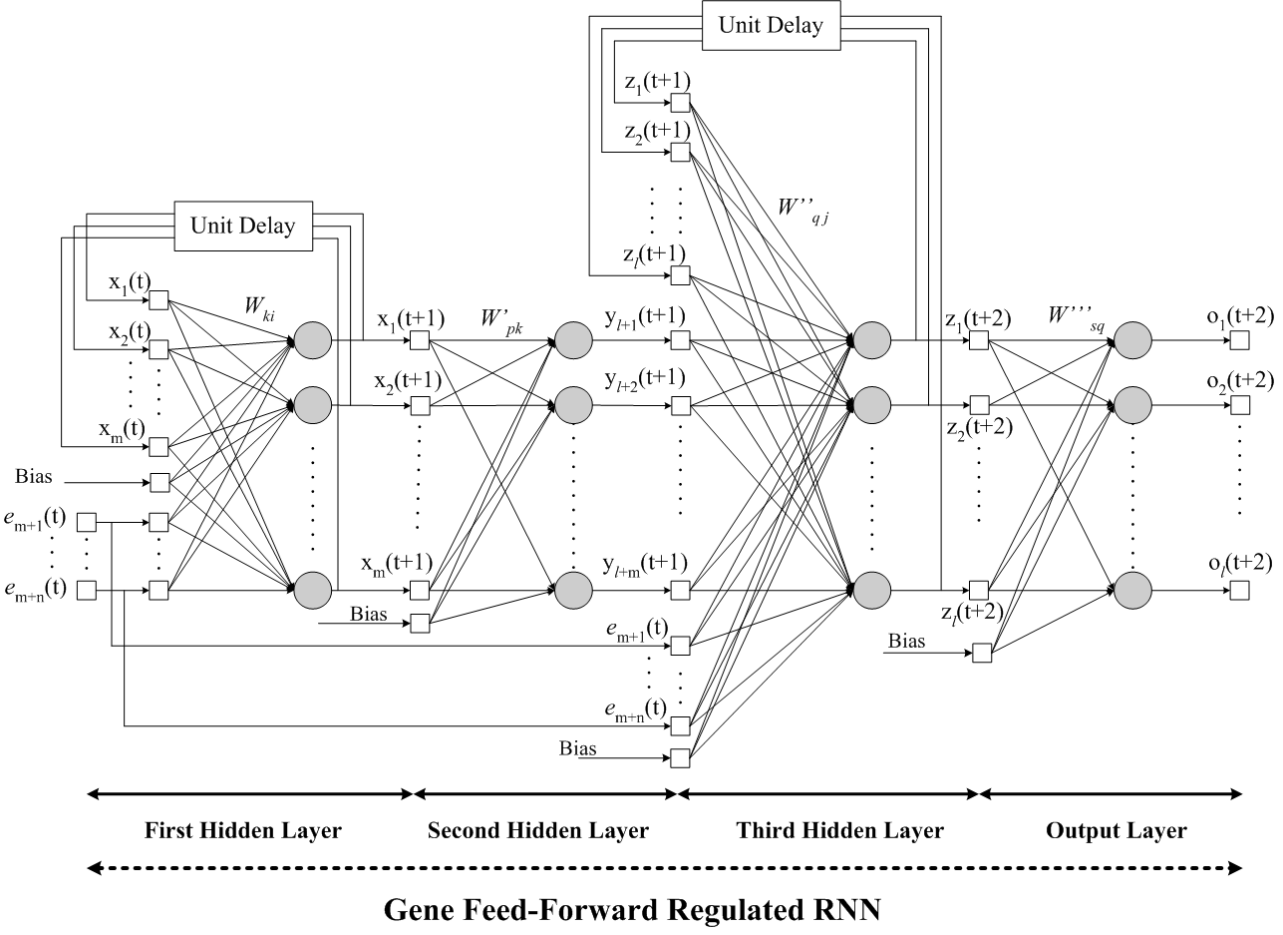
The RNN architecture for constructing feed-forward module.

Figure 1 demonstrates RNN architectures for various different kinds of regulatory modules used in our approach. The diagram shown in Figure 1(a) stands for the feed-forward motif, which contains the starter TF that controls the second TF in later time points and has the additional feature that both TFs have common target genes. It is of interest that the second TF is regulated by the principal TF at first and then controls other target genes together with the principal TF. This framework has an advantage of building simple regulatory pathways with the ordering of transcriptional influences and the time delay of this mechanism. In this approach, the modified RNN is altered from [1] to adjust to the nature of feed-forward transcriptional regulatory mechanisms. Figure 2 shows the detailed RNN architecture for constructing feed-forward regulatory modules. In this diagram, the expression level of a gene at a certain time point can be calculated by the weighted sum of the expression levels of all potential TFs in the network at a previous time point. For a time delay system, the models can be represented as:

xk(t+1)=ϕ(∑i=1mwkixi(t)+∑i=m+1m+nwkiei(t)+wkbB)     (3)
 MathType@MTEF@5@5@+=feaafiart1ev1aaatCvAUfKttLearuWrP9MDH5MBPbIqV92AaeXatLxBI9gBaebbnrfifHhDYfgasaacH8akY=wiFfYdH8Gipec8Eeeu0xXdbba9frFj0=OqFfea0dXdd9vqai=hGuQ8kuc9pgc9s8qqaq=dirpe0xb9q8qiLsFr0=vr0=vr0dc8meaabaqaciaacaGaaeqabaqabeGadaaakeaacqWG4baEdaWgaaWcbaGaem4AaSgabeaakiabcIcaOiabdsha0jabgUcaRiabigdaXiabcMcaPiabg2da9GGaciab=v9aQjabcIcaOmaaqahabaGaem4DaC3aaSbaaSqaaiabdUgaRjabdMgaPbqabaGccqWG4baEdaWgaaWcbaGaemyAaKgabeaakiabcIcaOiabdsha0jabcMcaPaWcbaGaemyAaKMaeyypa0JaeGymaedabaGaemyBa0ganiabggHiLdGccqGHRaWkdaaeWbqaaiabdEha3naaBaaaleaacqWGRbWAcqWGPbqAaeqaaOGaemyzau2aaSbaaSqaaiabdMgaPbqabaGccqGGOaakcqWG0baDcqGGPaqkcqGHRaWkcqWG3bWDdaWgaaWcbaGaem4AaSMaemOyaigabeaakiabdkeacjabcMcaPaWcbaGaemyAaKMaeyypa0JaemyBa0Maey4kaSIaeGymaedabaGaemyBa0Maey4kaSIaemOBa4ganiabggHiLdGccaWLjaGaaCzcamaabmaabaGaeG4mamdacaGLOaGaayzkaaaaaa@6BB3@

where the *e*_*i*_(*t*) is the gene expression level for the *i*^*th *^gene (*m *+ 1 ≤ *i *≤ *m *+ *n*, *n *is the number of beginning TFs), *w*_*ki *_(*m *+ 1 ≤ *i *≤ *m *+ *n*) represents the effect of the *k*^*th *^gene on the *i*^*th *^gene, and *B *is the bias. The *x*_*i*_(*t*) represents the *m *neurons in the hidden layer that are connected to the feedback nodes in the input layer, and the matrix *w*_*ki *_(1 ≤ *i *≤ *m*) represents the synaptic weights of the *m *neurons. A negative value of *w*_*ki *_represents the inhibition of the *k*^*th *^gene on the *i*^*th *^gene, while a positive value indicates the activation controls. The *ϕ*(), is a nonlinear sigmoidal function, usually in the form of *ϕ*(*z*) = 1/(1 + exp^-*z*^). The symbol *B *represents the bias, and the matrix *w*_*kb *_contains of the weights that represent bias terms applied to neuron 1 to *k*. The *x*_*k*_(*t *+ 1) represents the first hidden layer in the gene feed-forward regulated RNN, which stands for biological second ordered TFs (controlled by the starter TFs); the second and third hidden layers are listed as follows, respectively:

yp(t+1)=ϕ(∑k=1mw′pkxk(t+1)+w′pbB′)     (4)
 MathType@MTEF@5@5@+=feaafiart1ev1aaatCvAUfKttLearuWrP9MDH5MBPbIqV92AaeXatLxBI9gBaebbnrfifHhDYfgasaacH8akY=wiFfYdH8Gipec8Eeeu0xXdbba9frFj0=OqFfea0dXdd9vqai=hGuQ8kuc9pgc9s8qqaq=dirpe0xb9q8qiLsFr0=vr0=vr0dc8meaabaqaciaacaGaaeqabaqabeGadaaakeaacqWG5bqEdaWgaaWcbaGaemiCaahabeaakiabcIcaOiabdsha0jabgUcaRiabigdaXiabcMcaPiabg2da9GGaciab=v9aQjabcIcaOmaaqahabaGafm4DaCNbauaadaWgaaWcbaGaemiCaaNaem4AaSgabeaakiabdIha4naaBaaaleaacqWGRbWAaeqaaOGaeiikaGIaemiDaqNaey4kaSIaeGymaeJaeiykaKIaey4kaSIafm4DaCNbauaadaWgaaWcbaGaemiCaaNaemOyaigabeaakiqbdkeaczaafaGaeiykaKcaleaacqWGRbWAcqGH9aqpcqaIXaqmaeaacqWGTbqBa0GaeyyeIuoakiaaxMaacaWLjaWaaeWaaeaacqaI0aanaiaawIcacaGLPaaaaaa@56FB@

zq(t+2)=ϕ(∑j=1nw″qjej(t)+∑j=n+1n+mw″qjyj(t+1)+∑j=n+m+1n+m+lw″qjzj(t+1)+w″qbB″)     (5)
 MathType@MTEF@5@5@+=feaafiart1ev1aaatCvAUfKttLearuWrP9MDH5MBPbIqV92AaeXatLxBI9gBaebbnrfifHhDYfgasaacH8akY=wiFfYdH8Gipec8Eeeu0xXdbba9frFj0=OqFfea0dXdd9vqai=hGuQ8kuc9pgc9s8qqaq=dirpe0xb9q8qiLsFr0=vr0=vr0dc8meaabaqaciaacaGaaeqabaqabeGadaaakeaacqWG6bGEdaWgaaWcbaGaemyCaehabeaakiabcIcaOiabdsha0jabgUcaRiabikdaYiabcMcaPiabg2da9GGaciab=v9aQjabcIcaOmaaqahabaGafm4DaCNbayaadaWgaaWcbaGaemyCaeNaemOAaOgabeaakiabdwgaLnaaBaaaleaacqWGQbGAaeqaaOGaeiikaGIaemiDaqNaeiykaKIaey4kaSYaaabCaeaacuWG3bWDgaGbamaaBaaaleaacqWGXbqCcqWGQbGAaeqaaOGaemyEaK3aaSbaaSqaaiabdQgaQbqabaGccqGGOaakcqWG0baDcqGHRaWkcqaIXaqmcqGGPaqkaSqaaiabdQgaQjabg2da9iabd6gaUjabgUcaRiabigdaXaqaaiabd6gaUjabgUcaRiabd2gaTbqdcqGHris5aOGaey4kaSYaaabCaeaacuWG3bWDgaGbamaaBaaaleaacqWGXbqCcqWGQbGAaeqaaOGaemOEaO3aaSbaaSqaaiabdQgaQbqabaGccqGGOaakcqWG0baDcqGHRaWkcqaIXaqmcqGGPaqkcqGHRaWkcuWG3bWDgaGbamaaBaaaleaacqWGXbqCcqWGIbGyaeqaaOGafmOqaiKbayaacqGGPaqkaSqaaiabdQgaQjabg2da9iabd6gaUjabgUcaRiabd2gaTjabgUcaRiabigdaXaqaaiabd6gaUjabgUcaRiabd2gaTjabgUcaRiabdYgaSbqdcqGHris5aaWcbaGaemOAaOMaeyypa0JaeGymaedabaGaemOBa4ganiabggHiLdGccaWLjaGaaCzcamaabmaabaGaeGynaudacaGLOaGaayzkaaaaaa@8B7C@

where the *w*'_*pk *_is a weight matrix, for 1 ≤ *k *≤ *m*, and *B*' is the bias for this hidden layer. As in Figure 2, the third hidden layer contains *e*_*i*_(*t*) and *y*_*p*_(*t *+ 1) to demonstrate the expression values of the starter TFs and TFs controlled by themselves, respectively, and to co-regulate their common target genes described as:

Os(t+2)=ϕ(∑q=1lw‴sqzq(t+2)+w‴sbB‴)     (6)
 MathType@MTEF@5@5@+=feaafiart1ev1aaatCvAUfKttLearuWrP9MDH5MBPbIqV92AaeXatLxBI9gBaebbnrfifHhDYfgasaacH8akY=wiFfYdH8Gipec8Eeeu0xXdbba9frFj0=OqFfea0dXdd9vqai=hGuQ8kuc9pgc9s8qqaq=dirpe0xb9q8qiLsFr0=vr0=vr0dc8meaabaqaciaacaGaaeqabaqabeGadaaakeaacqWGpbWtdaWgaaWcbaGaem4CamhabeaakiabcIcaOiabdsha0jabgUcaRiabikdaYiabcMcaPiabg2da9GGaciab=v9aQjabcIcaOmaaqahabaGafm4DaCNbaibadaWgaaWcbaGaem4CamNaemyCaehabeaakiabdQha6naaBaaaleaacqWGXbqCaeqaaOGaeiikaGIaemiDaqNaey4kaSIaeGOmaiJaeiykaKIaey4kaSIafm4DaCNbaibadaWgaaWcbaGaem4CamNaemOyaigabeaakiqbdkeaczaasaGaeiykaKcaleaacqWGXbqCcqGH9aqpcqaIXaqmaeaacqWGSbaBa0GaeyyeIuoakiaaxMaacaWLjaWaaeWaaeaacqaI2aGnaiaawIcacaGLPaaaaaa@570E@

It is logical to assume that the time stamp of target genes is marked as *t *+ 2, *t *+ 1 for controlled TFs, and time stamp *t *for the starter TFs. In transcriptional progresses, regulatory pathway follows the prescribed order to "turn-on" or "turn-off" some target genes, and that is why we design a gene feed-forward regulated RNN architecture to fit the characteristic of the regulatory module. In the problem of regulatory module inference, the goal is to recover the regulatory interactions *w*_*ki*_, *w*'_*pk*_, *w*''_*qj *_and *w*'''_*sq*_. The instantaneous sum of squared errors at time *t *is defined in terms of *E*(*t*) by

E(t)=12∑s=1l(Os(t)−ds(t))2     (7)
 MathType@MTEF@5@5@+=feaafiart1ev1aaatCvAUfKttLearuWrP9MDH5MBPbIqV92AaeXatLxBI9gBaebbnrfifHhDYfgasaacH8akY=wiFfYdH8Gipec8Eeeu0xXdbba9frFj0=OqFfea0dXdd9vqai=hGuQ8kuc9pgc9s8qqaq=dirpe0xb9q8qiLsFr0=vr0=vr0dc8meaabaqaciaacaGaaeqabaqabeGadaaakeaacqWGfbqrcqGGOaakcqWG0baDcqGGPaqkcqGH9aqpdaWcaaqaaiabigdaXaqaaiabikdaYaaadaaeWbqaaiabcIcaOiabd+eapnaaBaaaleaacqWGZbWCaeqaaOGaeiikaGIaemiDaqNaeiykaKIaeyOeI0Iaemizaq2aaSbaaSqaaiabdohaZbqabaGccqGGOaakcqWG0baDcqGGPaqkcqGGPaqkdaahaaWcbeqaaiabikdaYaaaaeaacqWGZbWCcqGH9aqpcqaIXaqmaeaacqWGSbaBa0GaeyyeIuoakiaaxMaacaWLjaWaaeWaaeaacqaI3aWnaiaawIcacaGLPaaaaaa@4E70@

The objective of the learning process is to minimize a cost function obtained by summing *E*(*t*) over all time *t*; that is,

ETotal=∑tE(t)     (8)
 MathType@MTEF@5@5@+=feaafiart1ev1aaatCvAUfKttLearuWrP9MDH5MBPbIqV92AaeXatLxBI9gBaebbnrfifHhDYfgasaacH8akY=wiFfYdH8Gipec8Eeeu0xXdbba9frFj0=OqFfea0dXdd9vqai=hGuQ8kuc9pgc9s8qqaq=dirpe0xb9q8qiLsFr0=vr0=vr0dc8meaabaqaciaacaGaaeqabaqabeGadaaakeaacqWGfbqrdaWgaaWcbaGaemivaqLaem4Ba8MaemiDaqNaemyyaeMaemiBaWgabeaakiabg2da9maaqafabaGaemyrauKaeiikaGIaemiDaqNaeiykaKcaleaacqWG0baDaeqaniabggHiLdGccaWLjaGaaCzcamaabmaabaGaeGioaGdacaGLOaGaayzkaaaaaa@414F@

which measures the deviation of network output *O*(*t*) from the measurement (the target) *d*(*t*). We use gradient descent to determine the weights of the network and the weights correction of this gene feed-forward regulated RNN in the training phase are as follows:

Δwki=−η∂E∂wki=−η⋅[∑t∑s(Os−ds)]⋅∂Os∂wki     (9)
 MathType@MTEF@5@5@+=feaafiart1ev1aaatCvAUfKttLearuWrP9MDH5MBPbIqV92AaeXatLxBI9gBaebbnrfifHhDYfgasaacH8akY=wiFfYdH8Gipec8Eeeu0xXdbba9frFj0=OqFfea0dXdd9vqai=hGuQ8kuc9pgc9s8qqaq=dirpe0xb9q8qiLsFr0=vr0=vr0dc8meaabaqaciaacaGaaeqabaqabeGadaaakeaacqqHuoarcqWG3bWDdaWgaaWcbaGaem4AaSMaemyAaKgabeaakiabg2da9iabgkHiTGGaciab=D7aOnaalaaabaGaeyOaIyRaemyraueabaGaeyOaIyRaem4DaC3aaSbaaSqaaiabdUgaRjabdMgaPbqabaaaaOGaeyypa0JaeyOeI0Iae83TdGMaeyyXIC9aamWaaeaadaaeqbqaamaaqafabaGaeiikaGIaem4ta80aaSbaaSqaaiabdohaZbqabaGccqGHsislcqWGKbazdaWgaaWcbaGaem4CamhabeaakiabcMcaPaWcbaGaem4CamhabeqdcqGHris5aaWcbaGaemiDaqhabeqdcqGHris5aaGccaGLBbGaayzxaaGaeyyXIC9aaSaaaeaacqGHciITcqWGpbWtdaWgaaWcbaGaem4CamhabeaaaOqaaiabgkGi2kabdEha3naaBaaaleaacqWGRbWAcqWGPbqAaeqaaaaakiaaxMaacaWLjaWaaeWaaeaacqaI5aqoaiaawIcacaGLPaaaaaa@6602@

Δw′pk=−η′∂E∂w′pk=−η′⋅[∑t∑s(Os−ds)]⋅∂Os∂w′pk     (10)
 MathType@MTEF@5@5@+=feaafiart1ev1aaatCvAUfKttLearuWrP9MDH5MBPbIqV92AaeXatLxBI9gBaebbnrfifHhDYfgasaacH8akY=wiFfYdH8Gipec8Eeeu0xXdbba9frFj0=OqFfea0dXdd9vqai=hGuQ8kuc9pgc9s8qqaq=dirpe0xb9q8qiLsFr0=vr0=vr0dc8meaabaqaciaacaGaaeqabaqabeGadaaakeaacqqHuoarcuWG3bWDgaqbamaaBaaaleaacqWGWbaCcqWGRbWAaeqaaOGaeyypa0JaeyOeI0ccciGaf83TdGMbauaadaWcaaqaaiabgkGi2kabdweafbqaaiabgkGi2kqbdEha3zaafaWaaSbaaSqaaiabdchaWjabdUgaRbqabaaaaOGaeyypa0JaeyOeI0Iaf83TdGMbauaacqGHflY1daWadaqaamaaqafabaWaaabuaeaacqGGOaakcqWGpbWtdaWgaaWcbaGaem4CamhabeaakiabgkHiTiabdsgaKnaaBaaaleaacqWGZbWCaeqaaOGaeiykaKcaleaacqWGZbWCaeqaniabggHiLdaaleaacqWG0baDaeqaniabggHiLdaakiaawUfacaGLDbaacqGHflY1daWcaaqaaiabgkGi2kabd+eapnaaBaaaleaacqWGZbWCaeqaaaGcbaGaeyOaIyRafm4DaCNbauaadaWgaaWcbaGaemiCaaNaem4AaSgabeaaaaGccaWLjaGaaCzcamaabmaabaGaeGymaeJaeGimaadacaGLOaGaayzkaaaaaa@6746@

Δw″qj=−η″∂E∂w″qj=−η″⋅[∑t∑s(Os−ds)]⋅∂Os∂w″qj     (11)
 MathType@MTEF@5@5@+=feaafiart1ev1aaatCvAUfKttLearuWrP9MDH5MBPbIqV92AaeXatLxBI9gBaebbnrfifHhDYfgasaacH8akY=wiFfYdH8Gipec8Eeeu0xXdbba9frFj0=OqFfea0dXdd9vqai=hGuQ8kuc9pgc9s8qqaq=dirpe0xb9q8qiLsFr0=vr0=vr0dc8meaabaqaciaacaGaaeqabaqabeGadaaakeaacqqHuoarcuWG3bWDgaGbamaaBaaaleaacqWGXbqCcqWGQbGAaeqaaOGaeyypa0JaeyOeI0ccciGaf83TdGMbayaadaWcaaqaaiabgkGi2kabdweafbqaaiabgkGi2kqbdEha3zaagaWaaSbaaSqaaiabdghaXjabdQgaQbqabaaaaOGaeyypa0JaeyOeI0Iaf83TdGMbayaacqGHflY1daWadaqaamaaqafabaWaaabuaeaacqGGOaakcqWGpbWtdaWgaaWcbaGaem4CamhabeaakiabgkHiTiabdsgaKnaaBaaaleaacqWGZbWCaeqaaOGaeiykaKcaleaacqWGZbWCaeqaniabggHiLdaaleaacqWG0baDaeqaniabggHiLdaakiaawUfacaGLDbaacqGHflY1daWcaaqaaiabgkGi2kabd+eapnaaBaaaleaacqWGZbWCaeqaaaGcbaGaeyOaIyRafm4DaCNbayaadaWgaaWcbaGaemyCaeNaemOAaOgabeaaaaGccaWLjaGaaCzcamaabmaabaGaeGymaeJaeGymaedacaGLOaGaayzkaaaaaa@674D@

Δw‴sq=−η‴∂E∂w‴sq=−η‴⋅[∑t∑s(Os−ds)]⋅∂Os∂w‴sq     (12)
 MathType@MTEF@5@5@+=feaafiart1ev1aaatCvAUfKttLearuWrP9MDH5MBPbIqV92AaeXatLxBI9gBaebbnrfifHhDYfgasaacH8akY=wiFfYdH8Gipec8Eeeu0xXdbba9frFj0=OqFfea0dXdd9vqai=hGuQ8kuc9pgc9s8qqaq=dirpe0xb9q8qiLsFr0=vr0=vr0dc8meaabaqaciaacaGaaeqabaqabeGadaaakeaacqqHuoarcuWG3bWDgaGeamaaBaaaleaacqWGZbWCcqWGXbqCaeqaaOGaeyypa0JaeyOeI0ccciGaf83TdGMbaibadaWcaaqaaiabgkGi2kabdweafbqaaiabgkGi2kqbdEha3zaasaWaaSbaaSqaaiabdohaZjabdghaXbqabaaaaOGaeyypa0JaeyOeI0Iaf83TdGMbaibacqGHflY1daWadaqaamaaqafabaWaaabuaeaacqGGOaakcqWGpbWtdaWgaaWcbaGaem4CamhabeaakiabgkHiTiabdsgaKnaaBaaaleaacqWGZbWCaeqaaOGaeiykaKcaleaacqWGZbWCaeqaniabggHiLdaaleaacqWG0baDaeqaniabggHiLdaakiaawUfacaGLDbaacqGHflY1daWcaaqaaiabgkGi2kabd+eapnaaBaaaleaacqWGZbWCaeqaaaGcbaGaeyOaIyRafm4DaCNbaibadaWgaaWcbaGaem4CamNaemyCaehabeaaaaGccaWLjaGaaCzcamaabmaabaGaeGymaeJaeGOmaidacaGLOaGaayzkaaaaaa@67C1@

where the *η*, *η*', *η*'' and *η*''' represent the learning rate. The first derivative of *O*_*s *_with respect to *w*_*ki*_, *w*'_*pk*_, *w*_*qj*_'' and *w*_*sq*_''' are listed as follows:

∂Os∂w‴sq(t+2)=ϕ(Ω)⋅(1−ϕ(Ω))⋅zq(t+2)     (13)
 MathType@MTEF@5@5@+=feaafiart1ev1aaatCvAUfKttLearuWrP9MDH5MBPbIqV92AaeXatLxBI9gBaebbnrfifHhDYfgasaacH8akY=wiFfYdH8Gipec8Eeeu0xXdbba9frFj0=OqFfea0dXdd9vqai=hGuQ8kuc9pgc9s8qqaq=dirpe0xb9q8qiLsFr0=vr0=vr0dc8meaabaqaciaacaGaaeqabaqabeGadaaakeaadaWcaaqaaiabgkGi2kabd+eapnaaBaaaleaacqWGZbWCaeqaaaGcbaGaeyOaIyRafm4DaCNbaibadaWgaaWcbaGaem4CamNaemyCaehabeaaaaGccqGGOaakcqWG0baDcqGHRaWkcqaIYaGmcqGGPaqkcqGH9aqpiiGacqWFvpGAcqGGOaakcqqHPoWvcqGGPaqkcqGHflY1cqGGOaakcqaIXaqmcqGHsislcqWFvpGAcqGGOaakcqqHPoWvcqGGPaqkcqGGPaqkcqGHflY1cqWG6bGEdaWgaaWcbaGaemyCaehabeaakiabcIcaOiabdsha0jabgUcaRiabikdaYiabcMcaPiaaxMaacaWLjaWaaeWaaeaacqaIXaqmcqaIZaWmaiaawIcacaGLPaaaaaa@5BF4@

∂Os∂w″qj(t+2)=∂Os∂zq⋅∂zq∂w″qj(t+2)=(ϕ(Ω)⋅(1−ϕ(Ω))⋅ϕ(Ψ)⋅ϕ(1−ϕ(Ψ))⋅ej(t),ϕ(Ω)⋅(1−ϕ(Ω))⋅ϕ(Ψ)⋅ϕ(1−ϕ(Ψ))⋅yj(t+1),ϕ(Ω)⋅(1−ϕ(Ω))⋅ϕ(Ψ)⋅ϕ(1−ϕ(Ψ))⋅zj(t+1), 1≤j≤nn+1≤j≤n+mn+m+1≤n+m+l     (14)
 MathType@MTEF@5@5@+=feaafiart1ev1aaatCvAUfKttLearuWrP9MDH5MBPbIqV92AaeXatLxBI9gBaebbnrfifHhDYfgasaacH8akY=wiFfYdH8Gipec8Eeeu0xXdbba9frFj0=OqFfea0dXdd9vqai=hGuQ8kuc9pgc9s8qqaq=dirpe0xb9q8qiLsFr0=vr0=vr0dc8meaabaqaciaacaGaaeqabaqabeGadaaakeaafaqaaeGabaaabaWaaSaaaeaacqGHciITcqWGpbWtdaWgaaWcbaGaem4CamhabeaaaOqaaiabgkGi2kqbdEha3zaagaWaaSbaaSqaaiabdghaXjabdQgaQbqabaaaaOGaeiikaGIaemiDaqNaey4kaSIaeGOmaiJaeiykaKIaeyypa0ZaaSaaaeaacqGHciITcqWGpbWtdaWgaaWcbaGaem4CamhabeaaaOqaaiabgkGi2kabdQha6naaBaaaleaacqWGXbqCaeqaaaaakiabgwSixpaalaaabaGaeyOaIyRaemOEaO3aaSbaaSqaaiabdghaXbqabaaakeaacqGHciITcuWG3bWDgaGbamaaBaaaleaacqWGXbqCcqWGQbGAaeqaaaaakiabcIcaOiabdsha0jabgUcaRiabikdaYiabcMcaPaqaaiabg2da9maabeaabaqbaeaabmqaaaqaaGGaciab=v9aQjabcIcaOiabfM6axjabcMcaPiabgwSixlabcIcaOiabigdaXiabgkHiTiab=v9aQjabcIcaOiabfM6axjabcMcaPiabcMcaPiabgwSixlab=v9aQjabcIcaOiabfI6azjabcMcaPiabgwSixlab=v9aQjabcIcaOiabigdaXiabgkHiTiab=v9aQjabcIcaOiabfI6azjabcMcaPiabcMcaPiabgwSixlabdwgaLnaaBaaaleaacqWGQbGAaeqaaOGaeiikaGIaemiDaqNaeiykaKIaeiilaWcabaGae8x1dOMaeiikaGIaeuyQdCLaeiykaKIaeyyXICTaeiikaGIaeGymaeJaeyOeI0Iae8x1dOMaeiikaGIaeuyQdCLaeiykaKIaeiykaKIaeyyXICTae8x1dOMaeiikaGIaeuiQdKLaeiykaKIaeyyXICTae8x1dOMaeiikaGIaeGymaeJaeyOeI0Iae8x1dOMaeiikaGIaeuiQdKLaeiykaKIaeiykaKIaeyyXICTaemyEaK3aaSbaaSqaaiabdQgaQbqabaGccqGGOaakcqWG0baDcqGHRaWkcqaIXaqmcqGGPaqkcqGGSaalaeaacqWFvpGAcqGGOaakcqqHPoWvcqGGPaqkcqGHflY1cqGGOaakcqaIXaqmcqGHsislcqWFvpGAcqGGOaakcqqHPoWvcqGGPaqkcqGGPaqkcqGHflY1cqWFvpGAcqGGOaakcqqHOoqwcqGGPaqkcqGHflY1cqWFvpGAcqGGOaakcqaIXaqmcqGHsislcqWFvpGAcqGGOaakcqqHOoqwcqGGPaqkcqGGPaqkcqGHflY1cqWG6bGEdaWgaaWcbaGaemOAaOgabeaakiabcIcaOiabdsha0jabgUcaRiabigdaXiabcMcaPiabcYcaSaaacqqGGaaifaqaceWabaaabaGaeGymaeJaeyizImQaemOAaOMaeyizImQaemOBa4gabaGaemOBa4Maey4kaSIaeGymaeJaeyizImQaemOAaOMaeyizImQaemOBa4Maey4kaSIaemyBa0gabaGaemOBa4Maey4kaSIaemyBa0Maey4kaSIaeGymaeJaeyizImQaemOBa4Maey4kaSIaemyBa0Maey4kaSIaemiBaWgaaaGaayjkaaaaaiaaxMaacaWLjaWaaeWaaeaacqaIXaqmcqaI0aanaiaawIcacaGLPaaaaaa@09E6@

∂Os∂w′pk(t+1)=∂Os∂zq⋅∂zq∂yj⋅∂yj∂w′pk(t+1)=ϕ(Ω)⋅(1−ϕ(Ω))⋅ϕ(Ψ)⋅(1−ϕ(Ψ))⋅ϕ(Λ)⋅(1−ϕ(Λ))⋅ϕ(Γ)⋅(1−ϕ(Γ))⋅xi(t+1)     (15)
 MathType@MTEF@5@5@+=feaafiart1ev1aaatCvAUfKttLearuWrP9MDH5MBPbIqV92AaeXatLxBI9gBaebbnrfifHhDYfgasaacH8akY=wiFfYdH8Gipec8Eeeu0xXdbba9frFj0=OqFfea0dXdd9vqai=hGuQ8kuc9pgc9s8qqaq=dirpe0xb9q8qiLsFr0=vr0=vr0dc8meaabaqaciaacaGaaeqabaqabeGadaaakeaafaqaaeGabaaabaWaaSaaaeaacqGHciITcqWGpbWtdaWgaaWcbaGaem4CamhabeaaaOqaaiabgkGi2kqbdEha3zaafaWaaSbaaSqaaiabdchaWjabdUgaRbqabaaaaOGaeiikaGIaemiDaqNaey4kaSIaeGymaeJaeiykaKIaeyypa0ZaaSaaaeaacqGHciITcqWGpbWtdaWgaaWcbaGaem4CamhabeaaaOqaaiabgkGi2kabdQha6naaBaaaleaacqWGXbqCaeqaaaaakiabgwSixpaalaaabaGaeyOaIyRaemOEaO3aaSbaaSqaaiabdghaXbqabaaakeaacqGHciITcqWG5bqEdaWgaaWcbaGaemOAaOgabeaaaaGccqGHflY1daWcaaqaaiabgkGi2kabdMha5naaBaaaleaacqWGQbGAaeqaaaGcbaGaeyOaIyRafm4DaCNbauaadaWgaaWcbaGaemiCaaNaem4AaSgabeaaaaGccqGGOaakcqWG0baDcqGHRaWkcqaIXaqmcqGGPaqkaeaacqGH9aqpiiGacqWFvpGAcqGGOaakcqqHPoWvcqGGPaqkcqGHflY1cqGGOaakcqaIXaqmcqGHsislcqWFvpGAcqGGOaakcqqHPoWvcqGGPaqkcqGGPaqkcqGHflY1cqWFvpGAcqGGOaakcqqHOoqwcqGGPaqkcqGHflY1cqGGOaakcqaIXaqmcqGHsislcqWFvpGAcqGGOaakcqqHOoqwcqGGPaqkcqGGPaqkcqGHflY1cqWFvpGAcqGGOaakcqqHBoatcqGGPaqkcqGHflY1cqGGOaakcqaIXaqmcqGHsislcqWFvpGAcqGGOaakcqqHBoatcqGGPaqkcqGGPaqkcqGHflY1cqWFvpGAcqGGOaakcqqHtoWrcqGGPaqkcqGHflY1cqGGOaakcqaIXaqmcqGHsislcqWFvpGAcqGGOaakcqqHtoWrcqGGPaqkcqGGPaqkcqGHflY1cqWG4baEdaWgaaWcbaGaemyAaKgabeaakiabcIcaOiabdsha0jabgUcaRiabigdaXiabcMcaPaaacaWLjaGaaCzcamaabmaabaGaeGymaeJaeGynaudacaGLOaGaayzkaaaaaa@B8AC@

∂Os∂wki(t)=∂Os∂zq⋅∂zq∂yj⋅∂yj∂xk⋅∂xk∂wki(t)={ϕ(Ω)⋅(1−ϕ(Ω))⋅ϕ(Ψ)⋅(1−ϕ(Ψ))⋅ϕ(Λ)⋅(1−ϕ(Λ))⋅ϕ(Γ)⋅(1−ϕ(Γ))⋅xi(t),ϕ(Ω)⋅(1−ϕ(Ω))⋅ϕ(Ψ)⋅(1−ϕ(Ψ))⋅ϕ(Λ)⋅(1−ϕ(Λ))⋅ϕ(Γ)⋅(1−ϕ(Γ))⋅ei(t), 1≤i≤nn+1≤i≤n+m     (16)
 MathType@MTEF@5@5@+=feaafiart1ev1aaatCvAUfKttLearuWrP9MDH5MBPbIqV92AaeXatLxBI9gBaebbnrfifHhDYfgasaacH8akY=wiFfYdH8Gipec8Eeeu0xXdbba9frFj0=OqFfea0dXdd9vqai=hGuQ8kuc9pgc9s8qqaq=dirpe0xb9q8qiLsFr0=vr0=vr0dc8meaabaqaciaacaGaaeqabaqabeGadaaakeaafaqaaeGabaaabaWaaSaaaeaacqGHciITcqWGpbWtdaWgaaWcbaGaem4CamhabeaaaOqaaiabgkGi2kabdEha3naaBaaaleaacqWGRbWAcqWGPbqAaeqaaaaakiabcIcaOiabdsha0jabcMcaPiabg2da9maalaaabaGaeyOaIyRaem4ta80aaSbaaSqaaiabdohaZbqabaaakeaacqGHciITcqWG6bGEdaWgaaWcbaGaemyCaehabeaaaaGccqGHflY1daWcaaqaaiabgkGi2kabdQha6naaBaaaleaacqWGXbqCaeqaaaGcbaGaeyOaIyRaemyEaK3aaSbaaSqaaiabdQgaQbqabaaaaOGaeyyXIC9aaSaaaeaacqGHciITcqWG5bqEdaWgaaWcbaGaemOAaOgabeaaaOqaaiabgkGi2kabdIha4naaBaaaleaacqWGRbWAaeqaaaaakiabgwSixpaalaaabaGaeyOaIyRaemiEaG3aaSbaaSqaaiabdUgaRbqabaaakeaacqGHciITcqWG3bWDdaWgaaWcbaGaem4AaSMaemyAaKgabeaaaaGccqGGOaakcqWG0baDcqGGPaqkaeaacqGH9aqpdaGabeqaauaabeqaceaaaeaaiiGacqWFvpGAcqGGOaakcqqHPoWvcqGGPaqkcqGHflY1cqGGOaakcqaIXaqmcqGHsislcqWFvpGAcqGGOaakcqqHPoWvcqGGPaqkcqGGPaqkcqGHflY1cqWFvpGAcqGGOaakcqqHOoqwcqGGPaqkcqGHflY1cqGGOaakcqaIXaqmcqGHsislcqWFvpGAcqGGOaakcqqHOoqwcqGGPaqkcqGGPaqkcqGHflY1cqWFvpGAcqGGOaakcqqHBoatcqGGPaqkcqGHflY1cqGGOaakcqaIXaqmcqGHsislcqWFvpGAcqGGOaakcqqHBoatcqGGPaqkcqGGPaqkcqGHflY1cqWFvpGAcqGGOaakcqqHtoWrcqGGPaqkcqGHflY1cqGGOaakcqaIXaqmcqGHsislcqWFvpGAcqGGOaakcqqHtoWrcqGGPaqkcqGGPaqkcqGHflY1cqWG4baEdaWgaaWcbaGaemyAaKgabeaakiabcIcaOiabdsha0jabcMcaPiabcYcaSaqaaiab=v9aQjabcIcaOiabfM6axjabcMcaPiabgwSixlabcIcaOiabigdaXiabgkHiTiab=v9aQjabcIcaOiabfM6axjabcMcaPiabcMcaPiabgwSixlab=v9aQjabcIcaOiabfI6azjabcMcaPiabgwSixlabcIcaOiabigdaXiabgkHiTiab=v9aQjabcIcaOiabfI6azjabcMcaPiabcMcaPiabgwSixlab=v9aQjabcIcaOiabfU5amjabcMcaPiabgwSixlabcIcaOiabigdaXiabgkHiTiab=v9aQjabcIcaOiabfU5amjabcMcaPiabcMcaPiabgwSixlab=v9aQjabcIcaOiabfo5ahjabcMcaPiabgwSixlabcIcaOiabigdaXiabgkHiTiab=v9aQjabcIcaOiabfo5ahjabcMcaPiabcMcaPiabgwSixlabdwgaLnaaBaaaleaacqWGPbqAaeqaaOGaeiikaGIaemiDaqNaeiykaKIaeiilaWcaaiabbccaGuaabiqaceaaaeaacqaIXaqmcqGHKjYOcqWGPbqAcqGHKjYOcqWGUbGBaeaacqWGUbGBcqGHRaWkcqaIXaqmcqGHKjYOcqWGPbqAcqGHKjYOcqWGUbGBcqGHRaWkcqWGTbqBaaaacaGL7baaaaGaaCzcaiaaxMaadaqadaqaaiabigdaXiabiAda2aGaayjkaiaawMcaaaaa@2312@

where

Ω=∑q=1lw‴sqzq(t+2)     (17)
 MathType@MTEF@5@5@+=feaafiart1ev1aaatCvAUfKttLearuWrP9MDH5MBPbIqV92AaeXatLxBI9gBaebbnrfifHhDYfgasaacH8akY=wiFfYdH8Gipec8Eeeu0xXdbba9frFj0=OqFfea0dXdd9vqai=hGuQ8kuc9pgc9s8qqaq=dirpe0xb9q8qiLsFr0=vr0=vr0dc8meaabaqaciaacaGaaeqabaqabeGadaaakeaacqqHPoWvcqGH9aqpdaaeWbqaaiqbdEha3zaasaWaaSbaaSqaaiabdohaZjabdghaXbqabaGccqWG6bGEdaWgaaWcbaGaemyCaehabeaakiabcIcaOiabdsha0jabgUcaRiabikdaYiabcMcaPaWcbaGaemyCaeNaeyypa0JaeGymaedabaGaemiBaWganiabggHiLdGccaWLjaGaaCzcamaabmaabaGaeGymaeJaeG4naCdacaGLOaGaayzkaaaaaa@47BC@

Ψ=∑j=1nw″qjei(t)+∑j=n+1n+mw″qjyj(t+1)+∑j=n+m+1n+m+lw″qjzj(t+1)     (18)
 MathType@MTEF@5@5@+=feaafiart1ev1aaatCvAUfKttLearuWrP9MDH5MBPbIqV92AaeXatLxBI9gBaebbnrfifHhDYfgasaacH8akY=wiFfYdH8Gipec8Eeeu0xXdbba9frFj0=OqFfea0dXdd9vqai=hGuQ8kuc9pgc9s8qqaq=dirpe0xb9q8qiLsFr0=vr0=vr0dc8meaabaqaciaacaGaaeqabaqabeGadaaakeaacqqHOoqwcqGH9aqpdaaeWbqaaiqbdEha3zaagaWaaSbaaSqaaiabdghaXjabdQgaQbqabaGccqWGLbqzdaWgaaWcbaGaemyAaKgabeaakiabcIcaOiabdsha0jabcMcaPaWcbaGaemOAaOMaeyypa0JaeGymaedabaGaemOBa4ganiabggHiLdGccqGHRaWkdaaeWbqaaiqbdEha3zaagaWaaSbaaSqaaiabdghaXjabdQgaQbqabaGccqWG5bqEdaWgaaWcbaGaemOAaOgabeaakiabcIcaOiabdsha0jabgUcaRiabigdaXiabcMcaPaWcbaGaemOAaOMaeyypa0JaemOBa4Maey4kaSIaeGymaedabaGaemOBa4Maey4kaSIaemyBa0ganiabggHiLdGccqGHRaWkdaaeWbqaaiqbdEha3zaagaWaaSbaaSqaaiabdghaXjabdQgaQbqabaGccqWG6bGEdaWgaaWcbaGaemOAaOgabeaakiabcIcaOiabdsha0jabgUcaRiabigdaXiabcMcaPaWcbaGaemOAaOMaeyypa0JaemOBa4Maey4kaSIaemyBa0Maey4kaSIaeGymaedabaGaemOBa4Maey4kaSIaemyBa0Maey4kaSIaemiBaWganiabggHiLdGccaWLjaGaaCzcamaabmaabaGaeGymaeJaeGioaGdacaGLOaGaayzkaaaaaa@7C01@

Λ=∑k=1mw′pkxk(t+1)     (19)
 MathType@MTEF@5@5@+=feaafiart1ev1aaatCvAUfKttLearuWrP9MDH5MBPbIqV92AaeXatLxBI9gBaebbnrfifHhDYfgasaacH8akY=wiFfYdH8Gipec8Eeeu0xXdbba9frFj0=OqFfea0dXdd9vqai=hGuQ8kuc9pgc9s8qqaq=dirpe0xb9q8qiLsFr0=vr0=vr0dc8meaabaqaciaacaGaaeqabaqabeGadaaakeaacqqHBoatcqGH9aqpdaaeWbqaaiqbdEha3zaafaWaaSbaaSqaaiabdchaWjabdUgaRbqabaGccqWG4baEdaWgaaWcbaGaem4AaSgabeaakiabcIcaOiabdsha0jabgUcaRiabigdaXiabcMcaPaWcbaGaem4AaSMaeyypa0JaeGymaedabaGaemyBa0ganiabggHiLdGccaWLjaGaaCzcamaabmaabaGaeGymaeJaeGyoaKdacaGLOaGaayzkaaaaaa@476C@

Γ=∑i=1mwkixi(t)+∑i=m+1m+nwkiei(t)     (20)
 MathType@MTEF@5@5@+=feaafiart1ev1aaatCvAUfKttLearuWrP9MDH5MBPbIqV92AaeXatLxBI9gBaebbnrfifHhDYfgasaacH8akY=wiFfYdH8Gipec8Eeeu0xXdbba9frFj0=OqFfea0dXdd9vqai=hGuQ8kuc9pgc9s8qqaq=dirpe0xb9q8qiLsFr0=vr0=vr0dc8meaabaqaciaacaGaaeqabaqabeGadaaakeaacqqHtoWrcqGH9aqpdaaeWbqaaiabdEha3naaBaaaleaacqWGRbWAcqWGPbqAaeqaaOGaemiEaG3aaSbaaSqaaiabdMgaPbqabaGccqGGOaakcqWG0baDcqGGPaqkaSqaaiabdMgaPjabg2da9iabigdaXaqaaiabd2gaTbqdcqGHris5aOGaey4kaSYaaabCaeaacqWG3bWDdaWgaaWcbaGaem4AaSMaemyAaKgabeaakiabdwgaLnaaBaaaleaacqWGPbqAaeqaaOGaeiikaGIaemiDaqNaeiykaKcaleaacqWGPbqAcqGH9aqpcqWGTbqBcqGHRaWkcqaIXaqmaeaacqWGTbqBcqGHRaWkcqWGUbGBa0GaeyyeIuoakiaaxMaacaWLjaWaaeWaaeaacqaIYaGmcqaIWaamaiaawIcacaGLPaaaaaa@5C37@

Figures 1(b) and 1(c) demonstrate the RNN architectures for auto-regulate and multi-input motifs, respectively. The major difference between figure 1(b) and 1(c) is that the feedback comes from the output neurons or the hidden neurons of the multilayer perceptron to the input layer. Especially for figure 1(c), the single-input motif can be considered a subclass of the multi-input motif, and that is the reason we use the same RNN architecture to represent single-input and multi-input motifs.

**Figure 3:**
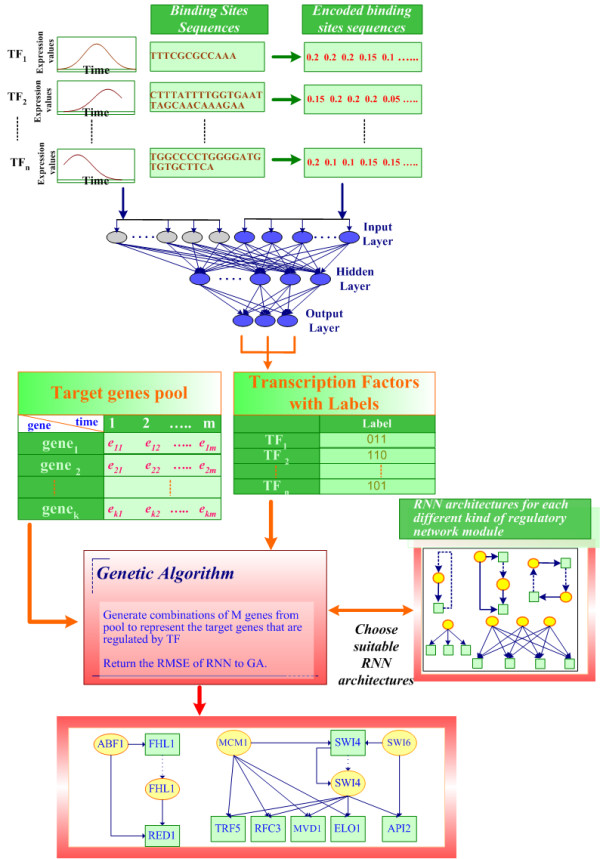
**The graphic algorithm of system procedures**. The whole system is implemented by Matlab 6.5. Notice that the RBF classifier is not designed for predicting novel TFs for yeast or human species. The main purpose of the RBF classifier is to group the kinds of categories that TFs belong to. As Figure shows, we input TFs into the system and the trained RBF classifier then can decide on the category according to the microarray expression values and transcription factor binding sites sequences of the TFs. This step also indicates that what kinds of RNN architecture are used in the following steps. The GA uses a standard random mutation and a standard binary representation with one point crossover. One TF may not only regulate one target gene, but may regulate several target genes simultaneously. To acquire the "good" combinations of target genes that are regulated by one or more common TFs, we select appropriate GA mutation and crossover operators to alter the chromosomes. One chromosome of the GA represents a number of genes taken from the full set of genes and is used by RNN to check how "good" the expression values of this combination of genes affected by particular transcription regulators are. On the other hand, the GA consists of populations of such chromosomes, and each chromosome is evaluated by the RNN for its fitted value to the given TFs. The choice of RNN architecture is according to the labels assigned by the RBF classifier. Furthermore, the final returned RNN output error (RMSE) is treated as a fitted value for some particular combinations of target genes. The stopping criterion includes not only the fitted value fit for some criteria but also the determination of the RNN selecting steps. In other words, the GA never stops until all appropriate RNN architectures are executed. In that case, each TF can choose suitable RNN architecture more than once, and find out a dissimilar set of target genes. After all TFs are run by this system procedures and output regulatory modules, the GA is then complete.

As described in previous section, we have various cell cycle-regulated TFs classified by RBF, and the next process is to find out the target genes. To resolve this problem, we must perform a global search for the optimal target genes which the GA produces. Hence, the neural computing method adopted in this paper combines the GA with the RNN architecture to form a hybrid system. We demonstrate the graphic algorithm in figure 3, and the procedures work as follows:

Input: various transcription factors

Output: regulatory modules with feedback and feed-forward control

Procedure:

For each transcription factor

BEGIN:

1. Randomly choose one TF *A *as the "input" gene, gene_A_.

2. If gene_A _is labeled as "010", then run steps 3 ~ 9 three times, one for the single-input motif RNN architecture, another for the multi-input motif RNN architecture, and the other for the feed-forward motif RNN architecture. It infers that, we have designed several RNN architectures for different motifs that are described in above section.

3. Use the genetic algorithm (GA) to generate combinations of M genes (gene_1_, gene_2_, ..., gene_m_) to represent the target genes that are regulated by gene_A_. Each combination is a chromosome. The initial set of combinations is composed of the initial population of chromosomes.

4. The training set, including the initial population of chromosomes and gene_A_, will consist of the microarray expression values for all the time points, and the initial population of chromosomes will be the target output for the RNN.

5. Execute the gradient descent algorithm on this training data via the RNN to determine the weights between the input genes and the output genes until stopping criterion is met.

6. Return the RMSE of RNN to GA. This is the fitted value for a particular chromosome.

7. Repeat 3, 4, and 5 for each chromosome.

8. Repeat steps 3~6 as a GA run, using crossover and mutation operators on all chromosomes to alter the choice of output gene combinations.

9. When some stopping criterion is met, the GA stops. Record the best chromosome and the weights of the RNN.

END

Change another TF (i.e. gene_B_), until no TFs are left.

When all the steps described above are completed, regulatory module structures can be derived to comprise the connections of the TFs and their target regulated genes. The parameters for the GA operators and RNN parameters are shown individually in Table 3.

**Table 3:** GA and RNN parameter settings

**Yeast Cell Cycle Data set**		**Human Cell Cycle Data set**	
**GA parameters**	**Values**	**GA parameters**	**Values**

Crossover	One Point, crossover rate (0.9)	Crossover	One Point, crossover rate (0.8)
Mutation	Random, mutation rate (0.05)	Mutation	Random, mutation rate (0.1)
Selector	Roulette Wheel	Selector	Roulette Wheel
Population Size	50 ~ 250	Population Size	100 ~ 250
Generations	100 ~ 1000	Generations	100 ~ 2500
**RNN parameters**	**Values**	**RNN parameters**	**Values**
Epochs	50 ~ 100	Epochs	100 ~ 200
Gradient descent	Standard	Gradient descent	Standard
Weight Update	Online	Weight Update	Online

## List of abbreviations

Genetic Algorithm (GA), Recurrent Neural Network (RNN), Transcription Factors (TFs), Radial Basis Function (RBF), Dynamic Bayesian Network (DBN)

## Authors' contributions

JHC and SYC participated in the design of this approach, analyzed the experimental results, and writing of the manuscript. SYC participated in the coding of the experiments and JHC participated in preparing the final draft of the manuscript. Both authors read and approved the final manuscript.

## Supplementary Material

Additional file 1 **Experimental results for human cell cycle and the biological support of the gene regulations**. Experimental results for human cell cycle and the biological support evidences of the gene regulations are listed in this file. Click here for file

Additional file 2 **The precision for yeast cell cycle**. The precision (TP/(TP+FP)) for yeast cell cycle data. Click here for file

Additional file 3 **Experiment for yeast cell cycle and the biological supports of the gene regulations**. Additional supporting analyses of yeast cell cycle and the biological supports for the article. Click here for file

Additional file 4 **Examples of TFs and their specific binding sites sequences**. The supplementary Tables A and B. We list examples of TFs and their binding sites sequences for human and yeast. Click here for file

Additional file 5 **The network motifs**. The network motifs used by this approach are described in additional file 5, including the auto-regulatory, feed-forward, single-input and multiple-input regulatory motifs. Click here for file

Additional file 6 **The architecture of RBF classifier**. The architecture of RBF classifier used by this approach is illustrated in this additional file. Click here for file
