# Supplementary material for: Modeling human cancer-related regulatory modules by GA-RNN hybrid algorithms
Source: BMC Bioinformatics. 2007 Mar 14;8:91. doi: 10.1186/1471-2105-8-91 (PMC1838431; doi:10.1186/1471-2105-8-91)
Supplement: Additional file 1 — Experimental results for human cell cycle and the biological support of the gene regulations. Experimental results for human cell cycle and the biological support evidences of the gene regulations are listed in this file. [file 1471-2105-8-91-S1.pdf]

Additional file 1 – Experimental results for human cell cycle and the biological support of the gene regulations

-Additional supporting analyses for the article: Jung-Hsien Chiang and Shih-Yi Chao: **Modeling human cancer-related regulatory modules by GA-RNN hybrid algorithms**

Discussions

Data from Whitfield *et al*. [35] is downloaded from the reference web site. The goal of human cell cycle analysis from Whitfield *et al.* is to identify >850 genes periodically expressed during the cell cycle, and to show that most of these genes have been previously associated with the proliferation of tumors during the human cell division cycle as well. We adopt this data set to construct cancer-related regulatory modules with feedback or feed-forward controlled target genes which are regulated by some specific TFs.

For the purpose of demonstration, we list experimental results for common regulators with [33]. Since yeast transcriptional regulatory mechanisms are more comprehended than Homo sapiens, we can confirm the accuracy of the connections within the regulatory modules provided by our system according to the published biological literatures which listed in the table below. We also provide simple explanations and descriptions for target genes. Some predicted results of target genes represent by boldface indicate the hypothetical targets. As listed in the table below, for instance, the E2F1 is a transcription factor of CCNA2, CDC25A, c-MYC, RFC3, p130, p107 and cycA, and E2F1 regulates the expression of these genes [4][5][7] [19][20].

Conclusions

Microarray technology has produced high-throughput data for exploring, analyzing, and understanding the phenomena of transcriptional regulatory mechanisms. We introduce a Genetic Algorithm-Recurrent Neural Network (GA-RNN) hybrid method for finding feed-forward regulated genes when given some transcription factors. It has proved that the gene regulations identified by GA-RNN have biological evidence supports, and it is a feasible approach to model human cancer-related regulatory modules.

Table S6 - Experimental results for human cell cycle and the biological support of the gene regulations

Regulator	Target predicted by [33] Expression data from Whitfield et al., 2002	Target predicted by our approach Expression data from Whitfield et al., 2002	Biological evidences *	Explanations and descriptions
E2F1	BUB1B, E2F1	E2F1 → E2F1	[1][24]	ChIP analysis demonstrated that E2F1 bound to E2F1 promoter chromatin in the G0 and early G1 cell cycle stage.[24]
		E2F1 → RB → cycA	[2][3][31]	E2F interacts with the CycA promoter and 5-prime UTR as demonstrated by chromatin immunoprecipitation (ChIP) assay. [31]
		E2F1 → RB →E2F3	[2][3][6]	The p16/RB/E2F regulatory pathway, which controls transit through of the G1 restriction point of the cell cycle, is one of the most frequent targets of genetic alterations in human cancer. Any of these alterations results in the deregulated expression of the transcription factor E2F, one of the key mediators of cell cycle progression. [2][3][6]
		E2F1→CDC6→CCNA	[2][18][30]	An interaction between E2F1 and CDC6 promoter was demonstrated by chromatin immunoprecipitation assay. Binding of E2F1 to the CDC6 promoter was reduced in MIF-/- EuMyc lymphomas. [30]
		RB, E2F3, CDC25A, CCNA2, P130, c-MYC, P107, P53, RFC3, cycA	[4][5] [7] [19][20][24]	E2F1 interacts with the CCNA2 promoter.[24] E2F1 is a transcription factor of CCNA2, CDC25A, c-MYC, RFC3, p130, p107 and cycA and regulates the expression of these genes. [4][5] [7] [19][20] An interaction between E2F1 and Cdc25A promoter chromatin was demonstrated by chromatin immunoprecipitation. [24]
		**RBL2, cyclinD1, CK2, TBL1, rbp6**	**hypothetical targets** *	

CDC25A	CCNE1, CDC20, CDC25A, STK15, BUB1B	CDC25A → CDC25A	[8]	Cdc25A is an important regulator of the G1/S transition but functions also in the mitotic phase of the human cell cycle.
		CCNE1, E2F1, E2F3, P130, MYC	[9] [10] [11][32]	An interaction between Myc and Cdc25a promoter was demonstrated by chromatin immunoprecipitation (ChIP) assay. [32] p130 interacts with the CDC25A promoter
		**CCNA1, CDK2**	**hypothetical targets** *	
CDC6	N/A	CDC6→ c-MYC→ORC2	[25][28]	In addition to its DNA replication activity, CDC6 also has a role as a transcriptional suppressor of c-Myc.[25]
		CDC6→PCAF→E2F1	[24]	[24] investigate the *p107*, *E2F-1*, *Cdc25A*, *Cdc6*, *B-myb*, *cyclin A*, and *Cdc2* promoters because each of these promoters has been implicated as a target of the E2F and pRB family on the basis of genetic and/or biochemical criteria.
		CDC6, E2F1, ORC2, c-MYC, P107, P130, RBL2, MCM7, E2F3,	[24][25] [26] [27] [29]	An interaction between p130 and Cdc6 promoter chromatin was demonstrated by chromatin immunoprecipitation.[24] some CDC6 protein is associated with the specific nuclear structure throughout the cell cycle and that major binding sites on chromatin differ between MCM and CDC6. CDC6 protein, a candidate regulator of MCM.[27]
		**NSEP1, PTHYA, OCD, GCN5, PCNA, ORC1C, AKAP8L, CCNA, NICD,**	**hypothetical targets** *	
PCAF	N/A	PCAF, p53, P21	[12]	Stimulation of p73-mediated transactivation by PCAF requires the HAT domain of PCAF and the p53-binding site within the p21 promoter. *In vivo*, coexpression of wild-type, but not HAT-deficient PCAF with p73 markedly increases p21 expression.
		**CP1C, BRCA2, CBF1, RAR-alpha, CK2,**	**hypothetical targets** *	

		**TBL1,**		
HDAC3	N/A	HDAC3, NCOR1, YY1, API2, TBL1, P53	[13][14] [15][21][22]	[13] shows that mammalian histone deacetylase (HDAC)-1, -2, and -3 are all capable of down-regulating p53 function. Down-regulation of p53 activity by HDACs is HDAC dosage-dependent, requires the deacetylase activity of HDACs, and depends on the region of p53 that is acetylated by p300/CREB-binding protein (CBP).
		**TGIF, TAB2, P50, GATA2, CK2, RBL2**	**hypothetical targets ***	
PCNA	CDC25B, CK2	RFC3, RB, P53, P50	[16] [17]	Lu et al. found that PCNA co-immunoprecipitated with human p50, as well as calf thymus DNA polymerase d heterodimer, but not with p125 alone, suggesting that PCNA directly interacts with p50 but not with p125.[17]
		**APC, COP1, PTK2, PTTG1**	**hypothetical targets ***	

* The gene names without boldface indicate the true positive (TP) targets that confirmed by searching in databases [34] or biological documents provided below.

References

1. Neuman E, Flemington EK, Sellers WR, Kaelin WG Jr: **Transcription of the E2F1 gene is rendered cell cycle dependent by E2F DNA-binding sites within its promoter**. *Mol. Cell. Biol.* 1994, **14**:6607-6615.
2. Stanelle J, Stiewe T, Theseling CC, Peter M, Putzer BM: **Gene Expression changes in response to E2F1 activation.** *Nucleic Acids Res.* 2002, **30**: 1859-1867.
3. Shan B, Chang CY, Jones D, Lee WH: **The transcription factor E2F1 mediates the autoregulation of RB gene expression.** *Mol. Cell. Biol.* 1994, **14**:299-309.
4. Vigo E, Muller H, Prosperini E, Hateboer G, Cartwright P, Moroni MC, Helin K: **CDC25A phosphatase is a target of E2F and is required for efficient E2F-induced S phase.** *Mol. Cell. Biol.* 1999, **19**:6379-6395.
5. Li W, Sanki A, Karim RZ, Thompson JF, Soon Lee C, Zhuang L, McCarthy SW, Scolyer RA: **The role of cell cycle regulatory proteins in the pathogenesis of melanoma.** *Pathology*. 2006, **38**:287-301.

6. Albino AP, Juan G, Traganos F, Reinhart L, Connolly J, Rose DP, Darzynkiewicz Z: **Cell cycle arrest and apoptosis of melanoma cells by docosahexaenoic acid: association with decreased pRb phosphorylation**. *Cancer Research* 2000, **60**:4139-4145.
7. Levine AJ: **p53, the Cellular Gatekeeper for Growth and Division.** *Cell* 1997, **88**: 323-331.
8. Kallstrom H, Lindqvist A, Pospisil V, Lundgren A, Rosenthal CK: **Cdc25A localisation and shuttling: characterisation of sequences mediating nuclear export and import.** *Experimental Cell Research* 2005**, 303**:89-100.
9. Liu D, Liao C, Wolgemuth DJ: **A Role for Cyclin A1 in the Activation of MPF and G2–M Transition during Meiosis of Male Germ Cells in Mice.** *Developmental Biology* 2000, **224**:388-400.
10. Crane R, Kloepfer A, Ruderman JV: **Requirements for the destruction of human Aurora-A.** *J Cell Sci* 2004, **117:** 5975-5983.
11. Balciunaite E, Spektor A, Lents NH, Cam H, Te Riele H, Scime A, Rudnicki MA, Young R, Dynlacht BD: **Pocket Protein Complexes Are Recruited to Distinct Targets in Quiescent and Proliferating Cells.** *Mol. And Cellular Biol.* 2005, **25**: 8166-8178.
12. Zhao LY, Liu Y, Bertos BR, Yang XJ, Liao D: **PCAF is a coactivator for p73-mediated transactivation.** *Oncogene* 2003, **22**: 8316-8329.
13. Juan LJ, Shia WJ, Chen MH, Yang WM, Seto E, Lin YS, Wu CW: **Histone deacetylases specifically down-regulate p53-dependent gene activation.** *Journal of Biological Chemistry* 2000, **27**: 20436-20443.
14. Ozawa Y, Towatari M, Tsuzuki S, Hayakawa F, Maeda T, Miyata Y, Tanimoto M, Saito H: **Histone deacetylase 3 associates with and represses the transcription factor GATA-2.** *Blood* 2001, **98**: 2116-2123.
15. Yoon HG, Chan DW, Huang ZQ, Li J, Fondell JD, Qin J, Wong J: **Purification and functional characterization of the human N-CoR complex: the roles of HDAC3, TBL1 and TBLR1**. *EMBO J.* 2003, **22**:1336-1346.
16. Ohta S**,** Shiomi Y, Sugimoto K, Obuse C, Tsurimoto T: **A proteomics approach to identify proliferating cell nuclear antigen (PCNA)-binding proteins in human cell lysates. Identification of the human CHL12/RFCs2-5 complex as a novel PCNA-binding protein.** *J. Biol. Chem.* 2002, **277**:40362-40367.
17. Lu X, Tan CK, Zhou JQ, You M, Carastro LM, Downey KM, So AG: **Direct interaction of proliferating cell nuclear antigen with the small subunit of DNA polymerase delta.** *J. Biol. Chem.* 2002, **277**:24340-24345.
18. Ohtani K, Tsujimoto A, Ikeda M, Nakamura M.: **Regulation of cell growth-dependent expression of mammalian CDC6 gene by the cell cycle transcription factor E2F.** *Oncogene* 1998, **17**:1777-1785.

19. Hiebert SW, Lipp M, Nevins JR: **E1A-dependent trans-activation of the human MYC promoter is mediated by the E2F factor.** Proc. Natl. Acad., Sci. 1989, **86**:3594-3598.
20. Sears R, Ohtan K, Nevins JR: **Identification of positively and negatively acting elements regulating expression of the E2F2 gene in response to cell growth signals.** *Mol. Biol. Cell* 1997, **17**:5227-5235.
21. Yang W M, Yao Y L, Sun J M, Davie J R, Seto E: **Isolation and characterization of cDNAs corresponding to an additional member of the human histone deacetylase gene family**. *J. Biol. Chem.* 1997, **272**:28001-28007.
22. Yang Y, Hwang CK, D'Souza U M, Lee S H, Junn E, Mouradian M M: **Three-amino acid extension loop homeodomain proteins Meis2 and TGIF differentially regulate transcription.** *J. Biol. Chem.* 2000, **275**:20734-20741.
23. Longworth MS, Wilson R, Laimins LA: **HPV31 E7 facilitates replication by activating E2F2 transcription through its interaction with HDACs.** *EMBO J* 2005, 24:1821-1830.
24. Takahashi Y, Rayman JB, Dynlacht BD: **Analysis of promoter binding by the E2F and pRB families in vivo: distinct E2F proteins mediate activation and repression**. *Genes Dev* 2000, 14:804-816.
25. Takayama M, Taira T, Iguchi-Ariga SM, Ariga H: **CDC6 interacts with c-Myc to inhibit E-box-dependent transcription by abrogating c-Myc/Max complex.** *FEBS Lett.* 2000, **477**:43-48.
26. Saha P, Chen J, Thome KC, Lawlis SJ, Hou ZH, Hendricks M, Parvin JD, Dutta A: **Human CDC6/Cdc18 associates with Orc1 and cyclin-cdk and is selectively eliminated from the nucleus at the onset of S phase**. *Mol Cell Biol.* 1998, 18:2758-67.
27. Fujita M, Yamada C, Goto H, Yokoyama N, Kuzushima K, Inagaki M, Tsurumi T: **Cell cycle regulation of human CDC6 protein. Intracellular localization, interaction with the human mcm complex, and CDC2 kinase-mediated hyperphosphorylation.** *J Biol Chem.* 1999, 274:25927-32.
28. Kneissl M, Putter V, Szalay AA, Grummt F: **Interaction and assembly of murine pre-replicative complex proteins in yeast and mouse cells.** *J Mol Biol.* 2003, **327**:111-28.
29. Petersen BO, Lukas J, Sorensen CS, Bartek J, Helin K: **Phosphorylation of mammalian CDC6 by cyclin A/CDK2 regulates its subcellular localization**. *EMBO J.* 1999, **18**:396-410.
30. Talos F, Mena P, Fingerle-Rowson G, Moll U, Petrenko O: **MIF loss impairs Myc-induced lymphomagenesis**. *Cell Death Differ* 2005, **12**:1319-1328.
31. Hernando E, Nahle Z, Juan G, Diaz-Rodriguez E, Alaminos M, Hemann M, Michel L, Mittal V, Gerald W, Benezra R, Lowe SW, Cordon-Cardo C: **Rb inactivation promotes**

genomic instability by uncoupling cell cycle progression from mitotic control. *Nature* 2004, **430**:797-802

32. Cairo S, De Falco F, Pizzo M, Salomoni P, Pandolfi PP, Meroni G: **PML interacts with Myc, and Myc target gene expression is altered in PML-null fibroblasts**. *Oncogene* 2005, **24**:2195-2203.
33. Li X, Rao S. Jiang W, Li C, Xiao Y, Guo Z, Zhang Q, Wang L, Du L, Li J, Li L, Zhang T. Wand QK: **Discovery of time-delayed gene regulatory networks based on temporal gene expression profiling**. *BMC Bioinformatics* 2006, **7**: 26-46.
34. Alfarano C, Andrade CE, Anthony K, Bahroos N, Bajec M, Bantoft K, Betel D, Bobechko B, Boutilier K, Burgess E, Buzadzija K, Cavero R, D'Abreo C, Donaldson I, Dorairajoo D, Dumontier MJ, Dumontier MR, Earles V, Farrall R, Feldman H, Garderman E, Gong Y, Gonzaga R, Grytsan V, Gryz E, Gu V, Haldorsen E, Halupa A, Haw R, Hrvojic A, Hurrell L, Isserlin R, Jack F, Juma F, Khan A, Kon T, Konopinsky S, Le V, Lee E, Ling S, Magidin M, Moniakis J, Montojo J, Moore S, Muskat B, Ng I, Paraiso JP, Parker B, Pintilie G, Pirone R, Salama JJ, Sgro S, Shan T, Shu Y, Siew J, Skinner D, Snyder K, Stasiuk R, Strumpf D, Tuekam B, Tao S, Wang Z, White M, Willis R, Wolting C, Wong S, Wrong A, Xin C, Yao R, Yates B, Zhang S, Zheng K, Pawson T, Ouellette BF, Hogue CW : **The Biomolecular Interaction Network Database and related tools 2005 update**. *Nucleic Acids Res.* 2005, **33**(Database issue):D418-24.
35. Whitfield ML, Sherlock G, Saldanha AJ, Murray JI, Ball CA, Alexander KE, Matese JC, Perou CM, Hurt MM, Brown PO, Botstein D: **Identification of Genes Periodically Expressed in the Human Cell Cycle and Their Expression in Tumors.** *Molecular Biology of the Cell* 2002, **13**: 1977-2000.
