# Supplementary material for: Modeling human cancer-related regulatory modules by GA-RNN hybrid algorithms
Source: BMC Bioinformatics. 2007 Mar 14;8:91. doi: 10.1186/1471-2105-8-91 (PMC1838431; doi:10.1186/1471-2105-8-91)
Supplement: Additional file 2 — The precision for yeast cell cycle. The precision (TP/(TP+FP)) for yeast cell cycle data. [file 1471-2105-8-91-S2.pdf]

Additional file 2 – The precision for yeast cell cycle

Discussions

Precision relates to the quality of an operation by which a result is obtained. As this is real-world data, the whole “real” gene connections of network are not available and, therefore, the accuracy of referred connections is determined by searching known gene connections in databases such as SGD [1] and BIND [2] for yeast cell cycle data. SGD is a database of the molecular biology and genetics of the yeast *Saccharomyces cerevisiae*. This database includes a variety of genomic and biological information, descriptions and classifications of yeast genes’ biological roles, molecular functions, and subcellular localizations. BIND is a database and stores full descriptions of interactions, molecular complexes or pathways for yeast and other species. As a result, we compare the referred interactions with those in known databases, and provide the precision in the table listed below.

The boldface gene names listed in table indicate the true positive (TP) targets that confirmed by searching in databases, and the gene names with red color represent the false positive (FP) targets. For the purpose of demonstration, we list experimental results of 8 regulators that [3] or [4] also provide. For instance, the experimental results for target genes of regulator MBP1 are provided by [4], but not [3]. Therefore, some fields represent N/A. The table also lists the precision of each regulator for our work and the average precision (TP/ (TP+FP)) is 80.14%.

Table 1 - The precision for yeast cell cycle

**Regulator** [3]	**Target predicted by [3]** expression data from Chou *et al*., 1998.	**Target predicted by [4]** [1] expression data from Spellman et al., 1998	**Target predicted by our approach** [2] expression data from Spellman et al., 1998	**Precision of our work Precision =TP/(TP+FP)**
SWI4	PCL1, CCN2, OCH1, HO, SWE1, GIN4, RNR1, PLB3, MNN1, NDD1, FKS1, CLB2, SPT21, RSR1, CWP1, BUD4, MBP1, PCL2, CLB6,	CDC28, SWI4, CLB2, CLB5	**SWI4**, **CDC6**, **APT2**, **TRF5**, **RFC3**, MVD1, ELO1, **TOS1**, ECM14, PSE1, **HO**, TOS7, **RSR1**, **TOS10**, **PLB3**, **HTA1**, PAN5, **PDR16, PCL1, CWP1, RFC3, CLB5**	72.73%
ACE2	SPO12	N/A	**KAR4**, ASH1, PCL9, PMA1, **FRS2**, **STE12, STE20, PHD1, AGP3, YAP1, GDH3, NCE4**	72.73%
MCM1	STE6, PIR3, CLN2, CLN3, GIN4, SIM1, MFA1	N/A	**SWI4**, **CDC46**, **TRF5**, **RFC3**, MVD1, **ELO1**, **CDC6**, API2, **PCL1, MSG5, MRD1, PIS1,** MTF2**, STE6, SAC7, TFC4, CLN3, UFD1, CDC46**	84.21%
FKH1	BUD8, HHF_1, ACE2, UTR2, SWI6	N/A	**BUD8**, **RHK1**, CHA1, **HOS3 MKK2**, **YMC2**, **RPN11**, **SVL3**, **PNP1**, **CLB4**, DYN1, **RHO4**, **SSO2**, **APE2**, **ACE2, ADH4, KIP2, SPC24, CHA1, DEM1, SUB2, ESP1, NUP145, ERS1**	91.67%
SWI6	HTB2, SIM1, YPR075, CDC6, AGA1, SPO12	SKP1, CLN2, SWI6	SPL2, ARO9, **PCL1**, **HPR5**, **TOK1**, AGP3, **MOT3, RFC3, CLB5, TOF1, MCD1, SWI2, TPS3, MRPL4, SWI4, ECM33, CLN1**	82.35%
SWI5	EGT2, MFA2, YLR463	CLB1, SWI5, SIC1	**TPS3**, CDC34, PST1, SIC1, **MRPL4, NMT1,** DPM1, PCL9, **NDD1, ASH1, SWI2, ASH1, FRS2, FAA3, KEX2, ENO1, DLD1, PDC6, PRY1, CST13, HSP150, GAT1, BUD9,** EGT2	79.12%
FKH2	GIC1, CLB4	N/A	SWI2, **ACE2**, ASH1, PCL9, PMA1, **FRS2**, ETG2,**SWI5, ACE2, KAR4, YMC2, SSO2, HOS3, MYO4, ADH4, KIP2, LTE1, RRM3, NCE4, UTH1**	75%
MBP1	N/A	MCM1, MBP1, SKP1	**CLB5**, **TOF1**, **MSH6**, **SWI4, ELO1, PCL1, KAR4, TRF5, RFC3,** YCK2, MCM2, **MCD1,**	83.33%
				Avg.=80.14%

[1] For the purpose of a numeric demonstration provided by [4], they chose 20 yeast cyclin genes. Therefore, some results are N/A.

[2] The boldface gene names indicate the true positive (TP) targets that confirmed by searching in databases [1][2], and the gene names with underlines represent the false positive (FP) targets. The average precision (TP/ (TP+FP)) is 80.14%.

[3] For the purpose of demonstration, we list experimental results of 8 regulators that [3] or [4] also provide. For example, the regulator ACE2, [3] provides the experimental results for target genes of ACE2, but [4] does not. Therefore, some fields listed above are represented N/A. We also provide the target genes predicted by our system for the common regulators, and search in database to verify.

References

1. Christie KR, Weng S, Balakrishnam R, Costanzo MC, Dolinski K, Dwight SS, Engel SR, Feierbach B, Fisk DG, Hirschman JE, Hong EL, Issel-Tarver L, Nash R, Sethuraman A, Starr B, Theesfeld CL, Andrada R, Binkley G, Dong Q, Lane C, Schroeder M, Botstein D, Cherry JM: **Saccharomyces Genome Database (SGD) provides tools to identify and analyze sequences from Saccharomyces cerevisiae and related sequences from other organisms.** *Nucleic Acids Res.* 2004, **32**: 311-314. [http://www.yeastgenome.org]
2. Alfarano C, Andrade CE, Anthony K, Bahroos N, Bajec M, Bantoft K, Betel D, Bobechko B, Boutilier K, Burgess E, Buzadzija K, Cavero R, D'Abreo C, Donaldson I, Dorairajoo D, Dumontier MJ, Dumontier MR, Earles V, Farrall R, Feldman H, Garderman E, Gong Y, Gonzaga R, Grytsan V, Gryz E, Gu V, Haldorsen E, Halupa A, Haw R, Hrvojic A, Hurrell L, Isserlin R, Jack F, Juma F, Khan A, Kon T, Konopinsky S, Le V, Lee E, Ling S, Magidin M, Moniakis J, Montojo J, Moore S, Muskat B, Ng I, Paraiso JP, Parker B, Pintilie G, Pirone R, Salama JJ, Sgro S, Shan T, Shu Y, Siew J, Skinner D, Snyder K, Stasiuk R, Strumpf D, Tuekam B, Tao S, Wang Z, White M, Willis R, Wolting C, Wong S, Wrong A, Xin C, Yao R, Yates B, Zhang S, Zheng K, Pawson T, Ouellette BF, Hogue CW : **The Biomolecular Interaction Network Database and related tools 2005 update**. *Nucleic Acids Res*. 2005, **33**(Database issue):D418-24.
3. Zou M, Conzen SD: **A new dynamic Bayesian network (DBN) approach for identifying gene regulatory networks from time course microarray data.** *Bioinformatics* 2005, **21**:71-79.
4. Li X, Rao S. Jiang W, Li C, Xiao Y, Guo Z, Zhang Q, Wang L, Du L, Li J, Li L, Zhang T. Wand QK: **Discovery of time-delayed gene regulatory networks based on temporal gene expression profiling**. *BMC Bioinformatics* 2006, **7**: 26-46.
