# Supplementary material for: Modeling human cancer-related regulatory modules by GA-RNN hybrid algorithms
Source: BMC Bioinformatics. 2007 Mar 14;8:91. doi: 10.1186/1471-2105-8-91 (PMC1838431; doi:10.1186/1471-2105-8-91)
Supplement: Additional file 3 — Experiment for yeast cell cycle and the biological supports of the gene regulations. Additional supporting analyses of yeast cell cycle and the biological supports for the article. [file 1471-2105-8-91-S3.pdf]

Additional file 3 – Experiment for yeast cell cycle and the biological support of the gene regulations

- Additional supporting analyses for the article: Jung-Hsien Chiang and Shih-Yi Chao:

Modeling human cancer-related regulatory modules by GA-RNN hybrid algorithms

Categorize yeast transcription factors by RBF

According to the results from [1], we observed that TFs can be divided into different kinds of motifs by their characteristics of regulation functions. It means that some TFs play critical roles in various motifs. Unlike Nachman *et al.* [2] and Qian *et al.*, [3], they predict regulatory modules by non-grouping TFs. We follow naive rules in yeast species, namely, we group TFs according to the primary function in advance according to the results from biological experiments. For instance, the regulator functions of ACE2 are suitable for the feed-forward loop, single input motif and multi-input motif. The SWI4 transcription factor belongs to the single input, multi-input, feed-forward, and auto-regulate motifs. For that reason, we regroup TFs into several catalogs and list some of them in Table 1, and find out the characteristic of classifying TFs by Radial Basis Function (RBF). In other words, learning RBF in our approach is in terms of the criterion for "the best fit" being measured in terms of microarray gene expressions and the TF binding sites sequences.

Construction of regulatory modules and results

After deciding the category of TFs, we use GA-RNN hybrid to construct the regulatory modules. One of the purposes of performing the yeast cell cycle data set experiment is to prove the capabilities of our GA-RNN hybrid algorithm. Since yeast transcriptional regulatory mechanisms are well-studied, we can confirm the accuracy of the connections within the regulatory modules provided by our system. That is, we examine the truth of the regulatory relationships found by searching in the published biological literature and by searching in KEGG, SGD and BIND databases [8][9][10]. We illustrate some experimental results with Table 2. Moreover, we also provide the precision for yeast cell cycle data in additional file 5.

Discussions

Figure 1 shows some examples of the estimated regulatory module connections with either feedback or feed-forward interactions. The analyses demonstrate that our proposed approach is capable of identification of significant regulatory modules and their corresponding regulatory interactions. Among the reconstruction regulatory modules, some have been confirmed by biological experiments. For instance, the GO annotations of ACE2 are “transcriptional activator activity” and “G1-specific transcription in mitotic cell cycle”. It has been proved that SWI5 and ACE2 are cell cycle regulated TFs that activate expression of early G1-specific genes in yeast. SWI5 and ACE2 also activate expression of a number of other genes expressed in the G1 phase of the cell cycle, including ASH1, CDC6, EGT2, PCL2, PCL9, RME1, and SIC1. The GO annotations of SWI4 are “G1/S transition of mitotic cell cycle” [4] and “cell cycle” [5]. They also confirm the efficiency of the reconstructions of regulatory modules. According to Sidorvoa *et al.* [6], SWI4 and SWI6 play crucial roles in yeast cell cycle. They analyze the protein complex of SWI4 and SWI6, which exhibit these two genes direct G1/S-specific transcription in yeast. Figure 6 also shows that the RAP1 and SWI4 are auto-regulate TFs, which are already improved biologically; FKH2 regulates cell cycle dependent expression of the CLB2 cluster of genes, including SWI5 and ACE2 [7].

Figure 1 - Some experimental results of regulatory modules for yeast cell cycle.

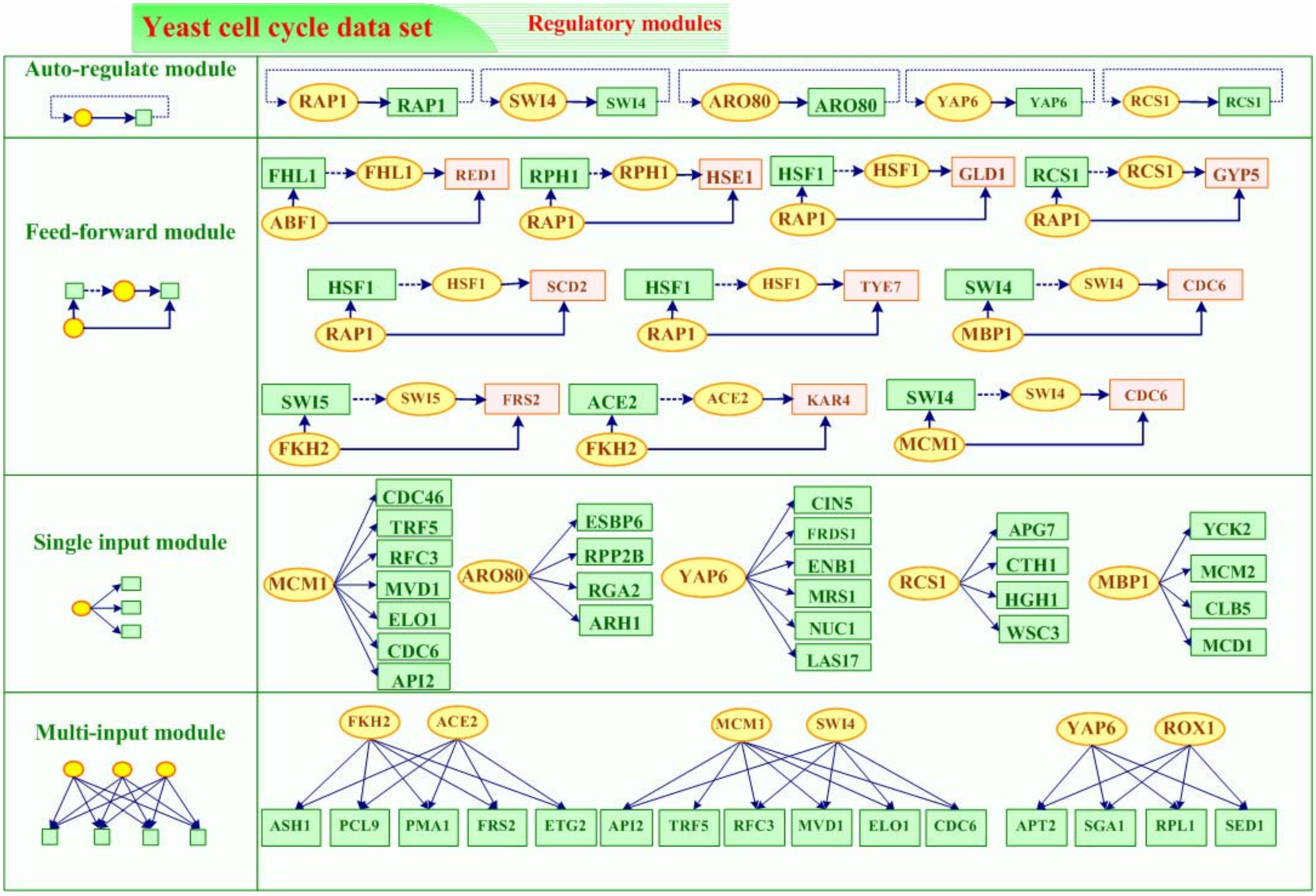

Circles indicate TFs and squares represent genes. Solid arrows indicate regulation relationships between TFs and their target genes

Table 1 - Some examples of regrouped TFs for yeast

Gene	Single Input	Multi-Input	Feed-forward	Auto-regulate	Encoded catalog number of RBF classifier
SWI4	√	√	√	√	001
ACE2	√	√	√		010
SUM1	√			√	100
ARO80				√	011
TAP6			√	√	101
REB1	√		√		110
FKH1	√	√			111
others					000

Table 2 - The experimental results of GA-RNN for yeast cell cycle data set

GA generations	Average RMSE	The minimum RMSE
100	3.44	0.24
200	1.17	0.22
400	1.58	0.22
600	0.85	0.21
800	0.80	0.19
1000	0.30	0.17

References

1. Lee TI, Rinaldi NJ, Robert F, Odom DT, Bar-Joseph Z, Gerber GK, Hannett NM, Harbison CT, Thompson CM, Simon I, Zeitlinger J, Jennings EG, Murry HL, Gordon DB, Ren B, Wyrick JJ, Tagne J, Volkert TL, Fraenkel E, Gifford DK, Young RA: **Transcriptional Regulatory Networks in Saccharomyces cerevisiae.** *Science* 2002, **298**: 799-804.
2. Nachman I, Regev A, Friedman N: **Inferring quantitative models of regulatory networks from expression data.** *Bioinformatics* 2004, **20**: 248-256.
3. Qian J, Lin J, Luscombe NM, Yu H, Gerstein M: **Prediction of regulatory networks: genome-wide identification of transcription factor targets from gene expression data.** *Bioinformatics* 2003, **19**: 1917-11926.
4. Munoz I, Simon E, Casals N, Clotet J, Arion J: **Identification of multicopy suppressors of cell cycle arrest at the G1-S transition in Saccharomyces cerevisiae.** *Yeast* 2003, **20**: 157-169.
5. Koch C, Nasmyth K: **Cell cycle regulated transcription in yeast.** *Molecular Cell Biology* 1994, **6**: 451-459.
6. Sidorvoa J, Breeden L: **Analysis of the SWI4/SWI6 protein complex, which directs G1/S-specific transcription in Saccharomyces cerevisiae**. *Molecular Cell Biology* 1993, **13**: 1069-1077.
7. Boros J, Lim FL, Darieva Z, Pic-Taylor A, Harman R, Morgan BA, Sharrocks A D: **Moecular determinants of the cell-cycle regulated Mcm1p -Fkh2p transcription factor complex.** *Nucleic Acids Res*. 2003, **31**: 2279-2288.
8. Kanehisa M, Goto S: **KEGG: kyoto encyclopedia of genes and genomes**. *Nucleic Acids Res* 2000, **28**(1):27-30.
9. Christie KR, Weng S, Balakrishnam R, Costanzo MC, Dolinski K, Dwight SS, Engel SR, Feierbach B, Fisk DG, Hirschman JE, Hong EL, Issel-Tarver L, Nash R, Sethuraman A, Starr B, Theesfeld CL, Andrada R, Binkley G, Dong Q, Lane C, Schroeder M, Botstein

D, Cherry JM: **Saccharomyces Genome Database (SGD) provides tools to identify and analyze sequences from Saccharomyces cerevisiae and related sequences from other organisms.** *Nucleic Acids Res.* 2004, **32**: 311-314. [http://www.yeastgenome.org]

10. Alfarano C, Andrade CE, Anthony K, Bahroos N, Bajec M, Bantoft K, Betel D, Bobechko B, Boutilier K, Burgess E, Buzadzija K, Cavero R, D'Abreo C, Donaldson I, Dorairajoo D, Dumontier MJ, Dumontier MR, Earles V, Farrall R, Feldman H, Garderman E, Gong Y, Gonzaga R, Grytsan V, Gryz E, Gu V, Haldorsen E, Halupa A, Haw R, Hrvojic A, Hurrell L, Isserlin R, Jack F, Juma F, Khan A, Kon T, Konopinsky S, Le V, Lee E, Ling S, Magidin M, Moniakis J, Montojo J, Moore S, Muskat B, Ng I, Paraiso JP, Parker B, Pintilie G, Pirone R, Salama JJ, Sgro S, Shan T, Shu Y, Siew J, Skinner D, Snyder K, Stasiuk R, Strumpf D, Tuekam B, Tao S, Wang Z, White M, Willis R, Wolting C, Wong S, Wrong A, Xin C, Yao R, Yates B, Zhang S, Zheng K, Pawson T, Ouellette BF, Hogue CW : **The Biomolecular Interaction Network Database and related tools 2005 update**. *Nucleic Acids Res*. 2005, **33**(Database issue):D418-24.
