# Supplementary material for: Modeling human cancer-related regulatory modules by GA-RNN hybrid algorithms
Source: BMC Bioinformatics. 2007 Mar 14;8:91. doi: 10.1186/1471-2105-8-91 (PMC1838431; doi:10.1186/1471-2105-8-91)
Supplement: Additional file 4 — Examples of TFs and their specific binding sites sequences. The supplementary Tables A and B. We list examples of TFs and their binding sites sequences for human and yeast. [file 1471-2105-8-91-S4.pdf]

Additional file 4 – Examples of TFs and their specific binding sites sequences

A. Human TFs and their specific binding sites sequences

**TF name : CDC6 (cell division cycle 6)**
GO annotations: GO:0007049, GO:0051301, GO:0008156,<br>*Binding sites sequences :* TTTCGCGCCAAA
**TF name : PCAF (p300/CBP-associated factor)**
GO annotations : GO:0003712, GO:0006355, GO:0008285<br>*Binding sites sequences :* CTTTATTTTGGTGAATTAGCAACAAAGAA
**TF name : CCNB2 (cyclin B2)**
GO annotations : GO:0000074<br>*Binding sites sequences :* TGGCCCCTGGGGATGTGTGCTTCA
**TF name : CDK7 (cyclin-dependent kinase 7)**
GO annotations : GO:0045893, GO:0051301, GO:0000079<br>*Binding sites sequences :* ATATTTTATATATACAGTATCT
**TF name : E2F1 (E2F transcription factor 1)**
GO annotations : GO:0000074, GO:0000080, GO:0000122, GO:0003700<br>*Binding sites sequences :* TTTCGCGC
**TF name : HDAC3 (histone deacetylase 3)**
GO annotations : GO:0000074, GO:0006355, GO:0007346, GO:0008134<br>*Binding sites sequences :* TTCCAGGCTCCTCACT

We collect known transcription factor binding sites sequences from ENSEMBL, TRANSFAC, SGD and YPD databases for human and yeast.

B. Examples of yeast TFs and their specific binding sites sequences

**TF name : ABF1 (YKL112W)**
GO annotations : GO:0006355, GO:0003700, GO:0045941<br>*Binding sites sequences :* RTCAYTNNNNACGW
**TF name : ACE2 (YLR131c)**
GO annotations : GO:0006355, GO:0016563, GO:0000114<br>*Binding sites sequences :* TGCTGGT
**TF name : FKH2 (YNL068C)**
GO annotations : GO:0000074, GO:0000116, GO:0003700, GO:0006355<br>*Binding sites sequences :* GTAAACA, RYMAAYA
**TF name : MCM1 (YMR043W)**
GO annotations : GO:0003700, GO:0003702, GO:0006355<br>*Binding sites sequences :* WTWCCYAAWNNGGTAA
**TF name : MSN4 (YKL062W)**
GO annotations : GO:0006355, GO:0003700<br>*Binding sites sequences :* AAGGGG
**TF name : MAC1 (YMR021C)**
GO annotations : GO:0006355, GO:0003700<br>*Binding sites sequences :* TTTGCTC
**TF name : RAP1 (YNL216W)**
GO annotations : GO:0003700, GO:0006355, GO:0045449<br>*Binding sites sequences :*WRMACCCATACAYY
**TF name : STE12 (YHR084w)**
GO annotations : GO:0003700, GO:0006355, GO:0007329<br>*Binding sites sequences :* ATGAAACA
**TF name : SWI4 (YER111c)**
GO annotations : GO:0003700, GO:0006355<br>*Binding sites sequences :* CACGAAAA

We collect known transcription factor binding sites sequences from ENSEMBL, TRANSFAC, SGD and YPD databases for human and yeast.
