# Supplementary material for: Modeling human cancer-related regulatory modules by GA-RNN hybrid algorithms
Source: BMC Bioinformatics. 2007 Mar 14;8:91. doi: 10.1186/1471-2105-8-91 (PMC1838431; doi:10.1186/1471-2105-8-91)
Supplement: Additional file 5 — The network motifs. The network motifs used by this approach are described in additional file 5, including the auto-regulatory, feed-forward, single-input and multiple-input regulatory motifs. [file 1471-2105-8-91-S5.pdf]

Additional file 5 - The network motifs

Circles represent TFs, and rectangles represent target genes. Solid arrows represent the regulatory relationship between TFs and their target genes. Dotted arrows indicate TFs and their product genes. Figure 1-(a) represents the auto-regulate motif, and it consists of a TF that binds to the promoter region of its own gene. Figure 1-(b) displays the Feed-forward motif, which contains a TF that controls a second TF and has the additional feature that both TFs bind to a common target gene. The multi-component loop motif, as illustrated in Figure 1-(c), consists of a regulatory circuit whose closure involves two or more TFs. The left two motifs exhibited in Figure 1-(d) and (e), including no feedback connections, are the single input motif and multi-input motif, respectively. We use these motifs as the basis of the regulatory modules constructed. In other words, the multi-layer RNN is modified by this approach to compose the various cancer-related regulatory modules, especially the feed-forward and feedback regulatory control.

Figure 1 - Transcriptional regulatory network motifs. This diagram is modified from [1].

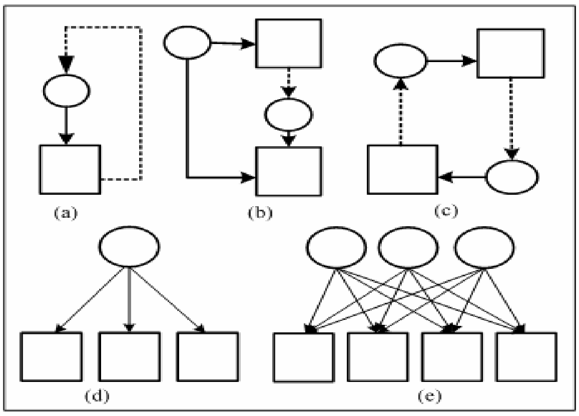

Reference

1. Lee TI, Rinaldi NJ, Robert F, Odom DT, Bar-Joseph Z, Gerber GK, Hannett NM, Harbison CT, Thompson CM, Simon I, Zeitlinger J, Jennings EG, Murry HL, Gordon DB, Ren B, Wyrick JJ, Tagne J, Volkert TL, Fraenkel E, Gifford DK, Young RA: **Transcriptional Regulatory Networks in Saccharomyces cerevisiae.** *Science* 2002, **298**: 799-804.
