# Supplementary material for: Modeling human cancer-related regulatory modules by GA-RNN hybrid algorithms
Source: BMC Bioinformatics. 2007 Mar 14;8:91. doi: 10.1186/1471-2105-8-91 (PMC1838431; doi:10.1186/1471-2105-8-91)
Supplement: Additional file 6 — The architecture of RBF classifier. The architecture of RBF classifier used by this approach is illustrated in this additional file. [file 1471-2105-8-91-S6.pdf]

Additional file 6 - The architecture of RBF classifier

The first kind of input nodes represent the original time points of gene expression values after being re-scaled between 0 and 1. The other kind of input nodes indicate the amino acids sequences of known transcription factor binding sites obtained from databases, which are also encoded into a number between 0 and 1. Let X represents the input layer vector, and G_i (where i =1, 2, …, n) represent the neurons in the hidden layer, which is a Gaussian kernel in the form:

$$G_i(X, \mu_i) = \exp(\frac{-1}{2\sigma_i^2} \| X - \mu_i \|^2) \quad (1)$$

where μ_i is a vector representing the center of i kernel and σ_i^2 is the corresponding variance. The output layer implements a weighted sum of hidden-output units:

$$F(X) = \sum_{i=1}^{m} w_i G(X, \mu_i) - \theta \quad \text{for } i = 1,2, \ldots, m \quad (2)$$

where w_i is the weight, and each corresponds individually to the connection between a hidden neuron and an output neuron. Later we use gradient descent to determine the weights of the network. At last, the vector θ represents biases. Finally, the outputs of RBF are “labels” that describe what categories the input TFs belong to.

Figure 1 - The architecture of RBF classifier

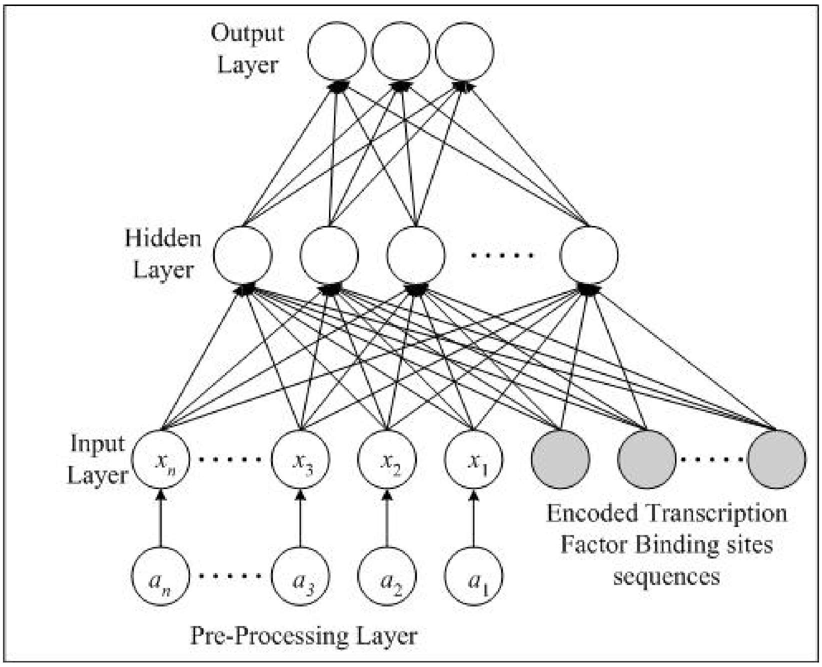

The shaded circles indicate the sequences of transcription factor binding sites.
